# Age and Primary Vaccination Background Influence the Plasma Cell Response to Pertussis Booster Vaccination

**DOI:** 10.3390/vaccines10020136

**Published:** 2022-01-18

**Authors:** Annieck M. Diks, Pauline Versteegen, Cristina Teodosio, Rick J. Groenland, Bas de Mooij, Anne-Marie Buisman, Alba Torres-Valle, Martín Pérez-Andrés, Alberto Orfao, Guy A. M. Berbers, Jacques J. M. van Dongen, Magdalena A. Berkowska, on behalf of the IMI-2 PERISCOPE Consortium

**Affiliations:** 1 Department of Immunology, Leiden University Medical Center, Albinusdreef 2, 2333 ZA Leiden, The Netherlands; A.M.Diks@lumc.nl (A.M.D.); C.I.Teodosio@lumc.nl (C.T.); R.J.Groenland@lumc.nl (R.J.G.); demooij.bas@gmail.com (B.d.M.); M.A.Berkowska@lumc.nl (M.A.B.); 2 Center for Infectious Disease Control, National Institute of Public Health and the Environment, 3721 MA Bilthoven, The Netherlands; pauline.versteegen@rivm.nl (P.V.); annemarie.buisman@rivm.nl (A.-M.B.); guy.berbers@rivm.nl (G.A.M.B.); 3 Cancer Research Centre (IBMCC, USAL-CSIC, CIBERONC CB16/12/00400), Institute for Biomedical Research of Salamanca (IBSAL), 37007 Salamanca, Spain; albatorresvalle@usal.es (A.T.-V.); mmmar@usal.es (M.P.-A.); orfao@usal.es (A.O.); 4 Department of Medicine and Cytometry Service (NUCLEUS Research Support Platform), University of Salamanca (USAL), 37007 Salamanca, Spain

**Keywords:** Tdap, flow cytometry, acellular pertussis vaccine (aP), whole-cell pertussis vaccine (wP), plasma cells, ELISpot, vaccine priming effect, plasma cell expansion

## Abstract

Pertussis is a vaccine-preventable disease caused by the bacterium *Bordetella pertussis*. Over the past years, the incidence and mortality of pertussis increased significantly. A possible cause is the switch from whole-cell to acellular pertussis vaccines, although other factors may also contribute. Here, we applied high-dimensional flow cytometry to investigate changes in B cells in individuals of different ages and distinct priming backgrounds upon administration of an acellular pertussis booster vaccine. Participants were divided over four age cohorts. We compared longitudinal kinetics within each cohort and between the different cohorts. Changes in the B-cell compartment were correlated to numbers of vaccine-specific B- and plasma cells and serum Ig levels. Expansion and maturation of plasma cells 7 days postvaccination was the most prominent cellular change in all age groups and was most pronounced for more mature IgG1+ plasma cells. Plasma cell responses were stronger in individuals primed with whole-cell vaccine than in individuals primed with acellular vaccine. Moreover, IgG1+ and IgA1+ plasma cell expansion correlated with FHA-, Prn-, or PT- specific serum IgG or IgA levels. Our study indicates plasma cells as a potential early cellular marker of an immune response and contributes to understanding differences in immune responses between age groups and primary vaccination backgrounds.

## 1. Introduction

Pertussis is a vaccine-preventable respiratory disease caused by the bacterium *Bordetella pertussis* (Bp). Since the introduction of the first pertussis vaccines in the 1940s and 1950s containing whole inactivated bacteria (whole-cell pertussis; wP), the incidence and mortality of pertussis have dramatically decreased [1]. However, the wP vaccine itself has a relatively high reactogenicity profile [2,3]. Therefore, from the early 1980s onwards, many (developed) countries started to replace the wP vaccine by an acellular pertussis (aP) vaccine, which had a more favorable reactogenicity profile [3,4]. In The Netherlands, this change took place on 1 January 2005. The aP vaccines contain purified Bp components, such as pertussis toxoid (PT), filamentous hemagglutinin (FHA), pertactin (Prn), and Fimbriae 2 and 3 (Fim2/3). In The Netherlands, the combined DTaP-IPV-Hib-HepB vaccine (providing protection against diphtheria, tetanus, pertussis, polio, *Haemophilus influenzae* type b (Hib), and hepatitis B) is used for primary vaccination. The booster vaccinations given at a later age are often Tdap vaccines [5].

Immune surveillance data have shown that despite high vaccination coverage in many countries, there has been an increase in pertussis cases in the past decennia [6,7]. This increase is not only seen in aP-using countries but was also reported in countries that primarily used wP vaccines at the time of investigation [6,8]. Several explanations for this increase have been proposed. First of all, improved awareness, surveillance, and diagnostics may increase the detection rate [9]. Furthermore, several new Bp strains have been described. These strains lack antigens present in the aP vaccine (such as FHA- or Prn-deficient strains), or PtxP3 strains that have adapted to suppress host immunity by producing higher levels of PT [10,11,12,13]. Lastly, there may be increased carriership within the population as well as faster waning of protective immunity in aP-primed individuals. Initial studies comparing the efficacy of aP vs. wP vaccines showed a similar short-term protection [14,15]. However, later long-term studies showed that protection lasted shorter when using aP vaccines [16,17,18,19]. Further, baboon models have shown that aP-induced immunity does not prevent transmission, immunity induced by wP vaccines leads to a faster clearance of bacteria, and immunity generated by infection prevents colonization [20]. These combined data point at the need for mucosal immunity to prevent or reduce colonization and carriership.

An improved vaccine, immunization program, and/or route of administration seem necessary to combat pertussis. This implies a need to first understand the mechanism underlying protection induced by aP and wP vaccines (their differences and similarities). So far, no true correlate of protection (neither serological nor cellular) has been established for pertussis, and this would greatly aid evaluation of newly developed vaccines. This is one of the pillars of the Innovative Medicines Initiative (IMI)-2 PERISCOPE Consortium (PERtussIS COrrelates of Protection Europe), which aims to increase the scientific understanding of pertussis-related immunity in humans, identify new biomarkers of protection, and generate technology and infrastructure for the future development of improved pertussis vaccines [21].

Several (recent) studies within and outside the IMI-2 PERISCOPE Consortium have shown that initial priming against pertussis (aP or wP vaccine) influences protection against disease as well as the immune response to (future) booster vaccinations [16,18,22,23,24,25]. For example, Hendrikx et al. found that in aP-primed children, antigen (Ag)-specific IgG4 serum levels were higher compared with those in wP-primed children [23]. Furthermore, Da Silva et al. showed that, even after receiving aP booster vaccinations, initial priming (wP or aP vaccine) determined the Ag-specific CD4 T-cell response [24]. Similarly, Lambert et al. showed that CD4 T cells isolated from recently aP-boosted individuals could be separated in a principal component analysis (PCA) view based on priming background. Here, an aP priming background resulted in a more Th2-related response compared to a wP priming background [22].

Neither vaccine-induced nor infection-induced immunity leads to lifelong protection against pertussis. Thus, the use of booster vaccinations later in life is a topic relevant for public health, as people with waned immunity can become carriers and, thus, a source of transmission. Moreover, older adults can be more vulnerable to severe disease outcomes [26]. Several studies have shown that aP boosters are effective and well-tolerated in (older) adults [27,28]. Recently, Versteegen et al. investigated the specific serological response to an aP booster vaccination in four cohorts of different ages and primary vaccination backgrounds in The Netherlands, Finland, and the UK (IMI-2 PERISCOPE study acronym: BERT, Booster pertussis vaccination study) [29]. Here, they found that all age cohorts showed a good response upon booster vaccination, with only limited differences between the different age cohorts for the Bp-specific IgG levels. However, Ag-specific serum IgA (both pre- and postvaccination) increased with age, likely caused by (mild) exposures to Bp over time.

Previous studies on influenza have shown that up to 80% of the circulating IgG plasma cells 7 days after vaccination can be vaccine-specific [30,31]. This, combined with the low numbers of circulating plasma cells at baseline (median counts <5 cells/μL [32]), implies that the plasma cell system is a relatively ‘clean’ system to monitor. Thus, flow cytometry may serve as a faster and less laborious approach to study vaccination-elicited plasma cells than typical Ag-specific approaches such as Enzyme-Linked Immunospot (ELISpot). Recently, our team used high-dimensional flow cytometry to investigate over-time cellular kinetics in 10 healthy (wP-primed) adults upon aP vaccination. We were able to demonstrate a clear expansion and maturation of plasma cells (especially IgG1+), and a strong correlation between IgG1+ memory B-cell expansion and the magnitude of the Ag-specific IgG serum response [33]. Here, we extended our exploratory study by the analysis of participants of different ages and different priming backgrounds after receiving an aP booster (Boostrix-IPV, GlaxoSmithKline (GSK), Wavre, Belgium). We included 48 individuals enrolled in the Dutch cohort used in the IMI-2 PERISCOPE–BERT study (periscope-project.eu/patients/study-2-bert/ accessed on 11 January 2022) at predefined time points, with the primary objective of describing the kinetics of circulating B-cell populations in four cohorts of different ages and with different priming backgrounds. 

## 2. Materials and Methods

### 2.1. Study Design and Sample Collection

This study comprises one of the exploratory objectives of the Dutch ‘BERT study’, which was initiated by the IMI-2 PERISCOPE Consortium. It was approved by Medical Research Ethics Committees United (MEC-U, NL60807.100.17-R17.039) and registered at the EU Clinical trial registry (EudraCT number 2016-003678-42). To be eligible for this study, participants had to (1) be generally healthy; (2) have no recent evidence of serious disease—i.e., requiring the use of immunosuppressive or immunomodulating medication—within the 3 months prior to inclusion; (3) received all regular vaccines according to Dutch National Immunization Program (www.rivm.nl/en/national-immunisation-programme, accessed on 18 November 2020) as appropriate for their age. An extensive description of the cohort and a complete overview of all inclusion and exclusion criteria was published recently by Versteegen and colleagues [29]. For convenience, an overview of inclusion and exclusion criteria specific for this study is provided in Supplementary Materials, Supplemental Table S1. A fraction of the participants of this aP vaccination study was subjected to additional exploratory analysis, such as mass cytometry, evaluation of mucosal antibodies, NGS, or in-depth flow cytometry. The exploratory substudy monitoring the fluctuations in circulating B-cell subsets at baseline and days 7, 14, and 28 after vaccination is discussed in this manuscript. Here, 48 individuals were selected from four cohorts of different ages and distinct priming backgrounds at infancy: children, 7–10 y/o, aP-primed; adolescents, 11–15 y/o, aP- or wP-primed (aiming for equal distribution of priming background); young adults, 20–34 y/o, wP-primed; older adults 60–70 y/o, in whom vaccination history was unknown (presumably, wP-primed or not vaccinated). Participants were selected from the study cohort of the overarching ‘BERT study’. For each cohort, the first 12 participants to be included for the BERT study were also included in this flow cytometric study. Dropouts were replaced by individuals that were included but had not yet started the BERT study. As we aimed for an equal distribution of priming background in the adolescent cohort, inclusion of participants for this cohort was guided by priming background and order of inclusion in the BERT cohort.

The study was conducted by the Spaarne Academy (Spaarne Hospital, Hoofddorp, The Netherlands). Written informed consent was obtained at the start of the study. Participants were vaccinated intramuscularly with the Boostrix-IPV vaccine after their first blood donation (baseline). Boostrix-IPV is a reduced-antigen Tdap–IPV booster vaccine, which contains diphtheria toxoid (Diph) (≥2.5 Limit of flocculation (Lf)); tetanus toxoid (Tet) (≥5 Lf); three Bordetella pertussis proteins, PT (8 µg), FHA (8 µg), Prn (2.5 µg); and inactivated poliovirus (Mahoney strain, 40 D-Antigen units (DU); MEF-1 strain, 8 DU; Saukett strain, 32 DU) and aluminum hydroxide as adjuvant [34]. Peripheral blood samples were collected in blood collection tubes using heparin as anticoagulant and in serum collection tubes at baseline, day 7, and day 28 after vaccination. An additional peripheral blood sample was taken at day 14 in participants aged 20–34 and aged 60–70. Individuals were excluded and replaced by a new participant if a blood sample at day 0 or 28 could not be obtained. 

### 2.2. Evaluation of Antigen-Specific Immunoglobulin Levels in Serum

Serological analysis was performed in all collected samples. Levels of IgG directed against Tet, PT, FHA, Prn, and Fim2/3, and levels of IgA directed against PT, FHA, Prn, and Fim2/3 were determined by multiplex immunoassay (MIA) at the Dutch National Institute for Public Health and the Environment (RIVM, The Netherlands) [35]. The serum antibody responses raised against Bp-antigens during the PERISCOPE–BERT study have been extensively discussed by Versteegen and colleagues [29].

### 2.3. Detection of Vaccine-Specific Antibody-Producing Plasma Cells and Memory B Cells

Analysis of numbers of IgG and IgA producing plasma cells and memory B cells was performed in the majority of the samples included in this study. B cells producing IgG directed against PT, FHA, Prn, and Tet, and B cells producing IgA direct against PT, FHA, and Prn were measured using ELISpot assay at the RIVM. This procedure has been described previously [36]. In short, peripheral blood mononuclear cells (PBMCs) were isolated using a density gradient. For antibody-producing plasma cells, PBMCs at day 7 postvaccination were directly transferred to Ag-coated ELISpot filter plates (duplicates). For the detection of vaccine-specific memory B cells, PBMCs collected at day 0 and day 28 were collected and stored at −135 °C. Thawed PBMCs were stimulated for 5 days using a culture medium containing CpG, IL-2, and IL-10. Next, cells were transferred to Ag-coated plates (duplicates). Numbers of Ag-specific antibody-producing cells—appearing as spots—were measured using an ImmunoSpot S6 Ultra-V analyzer (Cellular Technology Limited, Cleveland, OH). Uncoated wells filled with PBS served as negative control and were used to subtract background signal. Wells with a signal below the limit of quantification were set at 0.1 cell/10^5^ PBMCs. Cumulative IgG and IgA spot counts for all antigens measured were used for analyses.

### 2.4. Longitudinal Flow Cytometric Analysis of Circulating B-Cell Subsets 

All peripheral blood samples were subjected to high-throughput flow cytometric immunophenotyping of the B-cell compartment. Here, we used a recently developed BIGH-tube: the B-cell and plasma cell tube (BIGH) allows identification of >100 populations of B and plasma cells distinguished based on their maturation stage and expressed Ig subclasses [32,37] (Antibody panel and phenotypic description of the identified B-cell subsets: Supplemental Tables S2 and S3). 

Samples were processed according to the bulk lysis protocol using 10 × 10^6^ cells followed by intracellular staining, as described before [33] (protocols available on www.EuroFlow.org (accessed on 9 October 2017)), with the addition of membrane staining with CD45-AlexaFluor700. 

In short, based on the white blood cell count (as determined by an automated hematological analyzer (Sysmex XP-300, Sysmex Europe GmbH, Norderstedt, Germany)), one or multiple tubes were filled with up to 2 mL of blood, after which ammonium chloride was added up to a total volume of 50 mL. After a 15 min incubation at room temperature on a roller bank to lyse non-nucleated red blood cells, cells were washed, counted on a Sysmex XP-300, and pooled to a total of 10 × 10^6^ cells. Next, cells were stained with an antibody cocktail directed against surface markers for 30 min in the dark with the BIGH panel (Supplemental Table S2). This was followed by a cytoplasmic staining for intracellular Igs using the Fix & Perm reagent kit (Nordic MUbio, Susteren, The Netherlands) according to the manufacturer’s protocol. Finally, samples were washed and resuspended in PBS for immediate acquisition (or stored for max ~3 h at 4 °C).

For precise enumeration of cell numbers, we used Perfect-Count Microspheres™ (Cytognos) according to the EuroFlow SOP (protocol available on www.EuroFlow.org, accessed on 9 October 2017). In short, exactly 50 μL of well-mixed Perfect-Count Microspheres™ were added to exactly 50 μL of peripheral blood. Then, antibodies directed against CD19, CD3, and CD45 were added and the sample was incubated for 30 min in the dark. Next, 500 μL of NH4Cl was added and after 10 min incubation, samples were ready for immediate acquisition. Using this tube, we could identify and quantify total leukocytes and lymphocytes, B, T, and NK cells in each sample. All samples were acquired at the Flow cytometry Core Facility of LUMC, using a BD FACS LSR Fortessa 4L (BD Biosciences, San Jose, CA, USA) or a BD FACS LSR Fortessa X-20 flow cytometer (BD Biosciences), which were calibrated daily according to EuroFlow guidelines, as previously described [38,39].

### 2.5. Data Analysis and Statistics

To ensure objective data analysis and minimize operator-induced variability, all data were analyzed using the automated gating and identification (AGI) module of the Infinicyt software (Infinicyt™ Software v2.0, Cytognos). This AGI module makes use of clustering algorithms and comparison with fully annotated reference flow cytometry (FCS) data files of healthy individuals to assign clusters of events to a population [40]. Importantly, when there was no perfect fit for a cluster of events, this was marked as a ‘check’ population and the software indicated to which populations this cluster may correspond. These check events were assigned manually according to the proposed gating strategies for the BIGH panel (Supplemental Table S3) [32,37].

For visualization and statistical analysis, the GraphPad Prism 8.1.1 software (GraphPad, San Diego, CA, USA) was used. First, normality of distribution of major cell populations at baseline was evaluated using D’Agostino–Pearson Normality test. As not all major cell populations were normally distributed, a nonparametric approach was applied. To test longitudinal changes within each cohort, the Wilcoxon signed-rank test for paired samples was used. This was corrected for multiple testing by Bonferroni correction (in case of three sampling timepoints, *p* < 0.0167; in case of four sampling timepoints, *p* < 0.0083 was considered significant). To compare differences between the four cohorts at days 0, 7, and 28, the Kruskal–Wallis approach was used, followed by Dunn’s test. At day 14, only samples from the two adult cohorts were collected; therefore, the Mann–Whitney test was used instead of Kruskal–Wallis at day 14. This was corrected for multiple testing by Bonferroni correction (*p* < 0.0125 was considered significant). Correlations were determined using Spearman’s Ranking Correlation. Correlation coefficients with a *p* < 0.05 were considered significant. Within these significant correlations, correlation coefficients <0.6 or—in case of negative correlation, >−0.6—were considered weak correlations, whereas correlation coefficients >0.6 or <−0.6 were considered strong correlations. In Supplemental Tables S5 and S6, where many correlations were analyzed, we corrected for multiple testing by Bonferroni correction (*p* < 0.01 was considered significant). Lastly, in the comparison between aP- and wP-primed individuals (comparison of ratio over baseline at days 7 and 28), we performed Mann–Whitney, followed by Bonferroni correction (*p* < 0.025 was considered significant). 

## 3. Results

### 3.1. Study Cohorts

All participants enrolled in the study between October 2017 and March 2018. In total, 12 children (age: 7–10, aP-primed, m/f ratio: 6/6), 12 adolescents (age: 11–15, 7 individuals wP-primed, m/f ratio: 2/5;5 individuals aP-primed, m/f ratio: 4/1), 12 young adults (age 20–34, wP-primed, m/f ratio: 7/5), and 12 older adults (age 60–70, presumably wP-primed or not vaccinated, m/f ratio: 4/7) completed this study (as part of the PERISCOPE–BERT study). Three children who were initially enrolled dropped out and were replaced by three new participants. From all acquired samples, two baseline B-cell samples were lost due to technical problems (one child and one young adult). Finally, one older adult was excluded due to (potentially) clonal expansion of B cells and replaced by a new participant.

For most participants, the leukocyte, lymphocyte, T-cell, B-cell, and NK-cell counts at baseline were within the normal age-matched range (Table 1, Supplemental Table S4), or, in case of minor deviations, fell into the normal range at later time points [41,42]. Leukocytes, lymphocytes, and T cells remained mostly stable over the time of analysis. Although NK-cell numbers showed a minor decrease at day 28, this was most likely not related to the vaccination response (day 0 vs. 28, *p* ≤ 0.01; day 7 vs. 28, *p* ≤ 0.05, Supplemental Figure S1). There were no statistically significant differences in absolute leukocyte, lymphocyte, T-cell, and NK-cell counts at baseline between age cohorts. Thus, regarding the numbers of leukocytes, lymphocytes, B cells, T cells, and NK cells, our participants were healthy representatives of the general population.

### 3.2. Higher Counts of Naive B Cells and Plasma Cells in Children

B-cell numbers are known to decrease over time from an average of 1400 cells/μL in children <2 years to 200 cells/μL in adults [32,41]. This trend was also visible in our dataset, where children had more B cells than the adult groups (Table 1). This difference was mainly due to high numbers of pre-germinal center (naive) B cells in children. Although memory B-cell numbers were also higher in children than in adults (160 cells/μL in children vs. 85.7 cells/μL in young adults, n.s., and 160 cells/μL in children vs. 63.8 cells/μL in older adults, *p* ≤ 0.01), these differences were less prominent, and mainly restricted to IgG1+ and IgG3+ memory B cells. Finally, several plasma cell subsets were significantly more abundant in children than in adults (IgG1+, IgG3+, and IgD+ plasma cells), but due to their overall low frequencies, this did not have a major impact on total B-cell numbers. Limited differences in B-cell subset numbers were observed between adolescents and adults (Table 1). These differences were predominantly found in pre-germinal center (naive) B cells and individual plasma cell subsets. Thus, baseline cell numbers of B-cell subsets differed between the cohorts, which was in line with previously published data of age-matched individuals [32].

### 3.3. Expansion of Plasma Cells as the Most Prominent Cellular B-Cell Change after Vaccination

We have recently shown that aP booster vaccination in (wP-primed) adults triggers several cellular changes, of which the expansion of (predominantly) IgG1+ plasma cells at day 7 is most prominent [33]. Now, we set out to determine whether the same types of changes occur in vaccinated individuals, irrespective of age and primary vaccination background.

**Figure 1:**
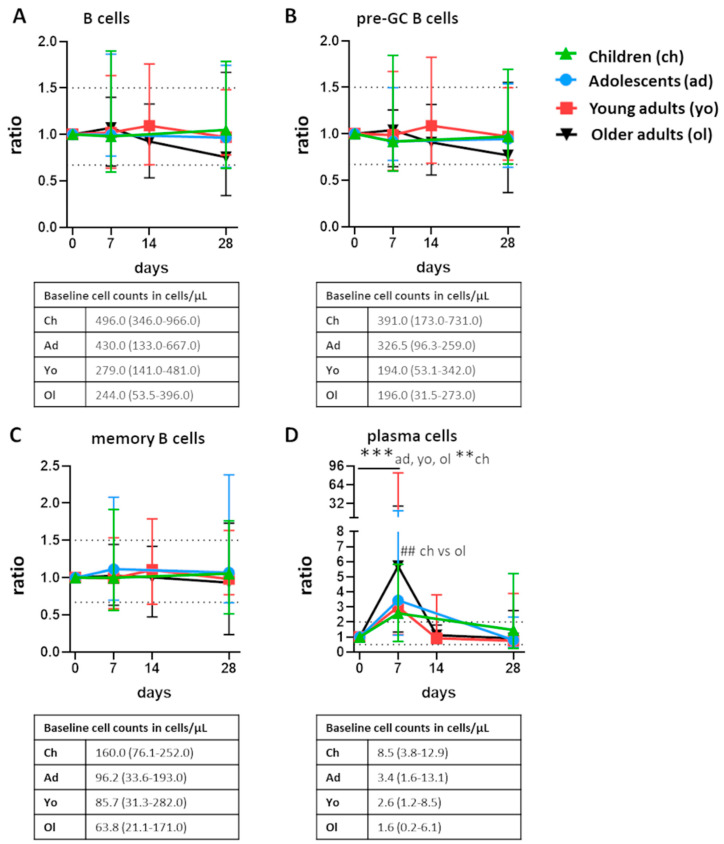
Postvaccination fluctuations of major B-cell subsets. Fluctuations of (**A**) total B cells (**B**) pre-germinal center (pre-GC) B cells, (**C**) memory B cells, and (**D**) plasma cells postvaccination presented as ratio over baseline (median, min-max). For total B cells, pre-GC B cells, and memory B cells, the dashed lines indicate a ratio over baseline of 0.67 and 1.5. For plasma cells, the dashed lines indicate a ratio over baseline of 0.5 and 2.0. Underneath each graph, the baseline cell counts per cohort are presented in cells/µL (median, min-max). To assess longitudinal changes within each cohort, Wilcoxon matched pair signed-rank test followed by Bonferroni correction was used. To test differences between cohorts at one timepoint, Kruskal–Wallis followed by Dunn’s test was used, with exception of the comparison at day 14. At day 14, only blood samples from the adult cohorts were collected; here, the Mann–Whitney test followed by Bonferroni correction was used. For longitudinal changes, only significant differences compared with baseline are shown. Significant longitudinal differences within a cohort are indicated as **, *p* ≤ 0.01; ***, *p* ≤ 0.001. Significant differences between cohorts at the same time point are indicated as ##, *p* ≤ 0.01.

Total B cells, pre-germinal center B cells, and memory B cells remained relatively stable and did not show any consistent fluctuations over time following vaccination. However, in all participants, plasma cells underwent significant expansion between baseline and day 7 (*p* ≤ 0.01 for children, and *p* ≤ 0.001 for adolescents and the adult cohorts, Figure 1). The magnitude of this expansion was highly similar in children, adolescents, and young adults (ratio to baseline: 2.6–3.4) and was significantly higher in older adults compared with children (ratio: 2.6 in children vs. 5.7 in older adults, *p* ≤ 0.01). Total plasma cell numbers returned to baseline at day 14 or, if day 14 was not measured, day 28 (*p* ≤ 0.001 for adolescents, young and older adults; in the children cohort, plasma cell counts were still slightly elevated at day 28). Thus, the expansion of plasma cells 7 days postvaccination was the most prominent change in all age groups.

### 3.4. Despite Individual Differences, Skewing towards IgG1+ Plasma Cell Responses in All Cohorts

**Figure 2:**
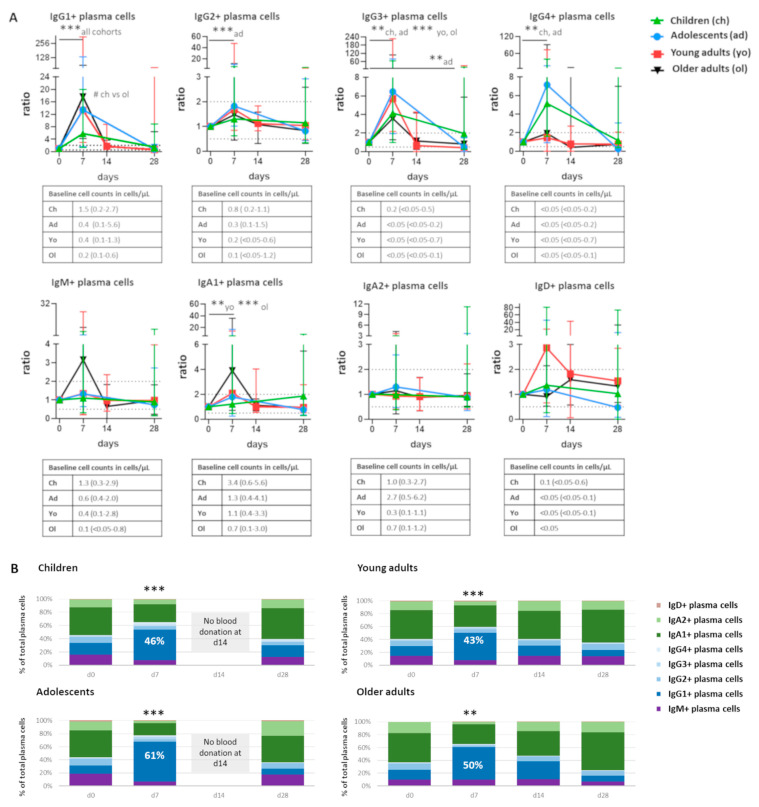
Individuals of all age groups underwent expansion of IgG1+ plasma cells at day 7 postvaccination. (**A**) Quantitative changes in plasma cells expressing different Ig subclasses, presented as ratio over baseline (median, min-max). Each symbol represents a median value with range. Dashed lines indicate a ratio over baseline of 0.5 and 2. Underneath each graph, the baseline cell counts per cohort are presented in cells/µL (median, min-max). Wilcoxon matched pair signed-rank test followed by Bonferroni correction was used to assess longitudinal differences within each cohort. Differences in ratio at day 7 between cohorts were assessed using Kruskal–Wallis followed by Dunn’s test. (**B**) Over-time distribution of plasma cells expressing different Ig subclasses. Median values for each population were used to construct the plots. Wilcoxon matched pair signed-rank test followed by Bonferroni correction was used to assess longitudinal differences in the percentage of IgG1+ cells in the total plasma cell compartment within each cohort. Differences in the percentage of IgG1+ cells in total plasma cell compartment between cohorts were assessed using Kruskal–Wallis followed by Dunn’s test but did not yield significant differences. For pediatric cohorts, no blood samples were collected at day 14; the Mann–Whitney test followed by Bonferroni correction was used. For longitudinal changes, only significant differences compared with baseline are shown. Significant longitudinal differences within a cohort are indicated as **, *p* ≤ 0.01; ***, *p* ≤ 0.001. Significant differences between cohorts at the same time point are indicated as #, *p* < 0.05. d = days after vaccination.

Cell expansion at day 7 was not equally pronounced in all plasma cell subsets. It was most prominent in IgG1+ plasma cells (ratio to baseline: 5.7–17.6, depending on cohort, *p* ≤ 0.001 in all cohorts, with a higher increase in older adults compared with children, *p* < 0.05). This was followed by IgG3+ plasma cells (ratio: 3.6–6.5, *p* ≤ 0.01 in children and adolescents, and *p* ≤ 0.001 in young and older adults, Figure 2A). IgG4+ plasma cells were significantly increased in children and adolescents (ratio: 5.1 and 7.2, respectively, *p* ≤ 0.01). IgG2+ plasma cells were significantly increased in adolescents only (ratio: 1.8, *p* ≤ 0.001). IgA1+ plasma cells were significantly increased in both adult cohorts (ratio: 2.1 and 3.9 in younger and older adults, *p* ≤ 0.01 and *p* ≤ 0.001, respectively). Although IgM+ plasma cells seemed to expand at day 7 in older adults, there was a large variation between individuals and the difference was not statistically significant.

Despite individual changes in response patterns between the cohorts, IgG1+ plasma cells were the most expanded subset. They constituted between 43% (young adults) and 61% (adolescents) of all plasma cells at the peak of expansion, while only at most 18% at baseline (Figure 2B). Although the ratio (to baseline) of plasma cells was often higher in adults than in children, children had higher baseline IgG1+ plasma cell numbers, and also higher IgG1+ plasma cell numbers at day 7 (median cell count at day 7: 7.64 cells/µL in children vs. 3.81 cells/µL in older adults, ns). Thus, the expansion of IgG1+ plasma cells was the most prominent in all cohorts. Both an increase over baseline (ratio) and in absolute cell numbers were observed.

### 3.5. Maturation of Plasma Cells over Time Following Vaccination 

**Figure 3:**
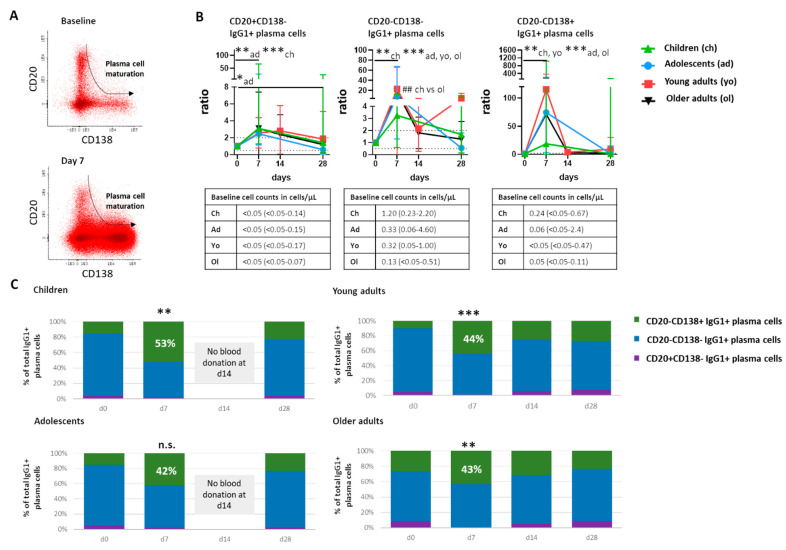
Over-time maturation of IgG1+ plasma cells. (**A**) Representative dot plots showing the phenotypical changes during plasma cell maturation. The top plot shows a baseline situation and the bottom plot shows a situation at day 7 postvaccination. Each dot represents an individual cell. The arrow indicates the direction of changes during maturation. (**B**) Over-time quantitative changes in IgG1+ plasma cells belonging to different maturation stages, presented as ratio over baseline (median, min-max). Dashed lines indicate a ratio over baseline of 0.5 and 2. Underneath each graph, a table shows the baseline cell counts of that population in cells/µL (median, min-max). (**C**) Over-time distribution of IgG1+ plasma cells representing different maturation stages with total IgG1+ plasma cells based on expression of CD20 and CD138. Median values for each population were used to construct the plots. Wilcoxon matched pair signed-rank test followed by Bonferroni correction was used to assess longitudinal differences in percentage of CD20-CD138+ cells in total IgG1+ plasma cells within each cohort. Differences in the percentage of CD20-CD138+ cells in total IgG1+ plasma cells between cohorts were assessed using Kruskal–Wallis followed by Dunn’s test but did not yield significant differences. For pediatric cohorts, no blood samples were collected at day 14; the Mann–Whitney test followed by Bonferroni correction was used. For longitudinal changes, only significant differences compared with baseline are shown. Significant longitudinal differences within a cohort are indicated as *, *p* < 0.05; **, *p* ≤ 0.01; ***, *p* ≤ 0.001. Significant differences between cohorts at the same time point are indicated as ##, *p* ≤ 0.01. d = days after vaccination.

Newly generated plasma cells migrate from germinal centers via the blood stream to become long-lived antibody-secreting plasma cells in the bone marrow and other peripheral tissues. Over time, they gradually lose expression of CD20 and gain expression of CD138 [43] (Figure 3A). We used this information to divide plasma cells into consecutive maturation stages and to trace their maturation over time after booster vaccination.

Plasma cells representing all maturation stages were expanded at day 7 (Supplemental Figure S2 for total plasma cells, Figure 3 for IgG1+ plasma cells). This expansion was limited in the least mature CD20+CD138- plasma cells (ratio to baseline: up to 3.1 in IgG1+ plasma cells in children, Figure 3B), clearer in intermediate CD20-CD138- plasma cells (ratio to baseline: up to 22.7 in IgG1+ plasma cells in young adults, in all cohorts *p* ≤ 0.01 or *p* ≤ 0.001), and most prominent in the most mature CD20-CD138+ plasma cells (ratio to baseline: up to of 115.6 in young adults, in all cohorts *p* ≤ 0.01 or *p* ≤ 0.001). Similar to what was observed for total plasma cells, expansion of IgG1+ plasma cells belonging to different maturation stages was least prominent in children. In all cohorts, a significant increase in the percentage of the most mature plasma cells was observed, except for the adolescent cohort, showing just a trend. At day 7 postvaccination, most mature plasma cells constituted between 42% and 53% of IgG1+ plasma cells (Figure 3C). Thus, the expansion of plasma cells at day 7 postvaccination was accompanied by a shift towards a more mature plasma cell phenotype in all cohorts.

As an expansion of IgA1+ plasma cells was observed in the adult cohorts, we evaluated maturation of IgA1+ plasma cells at the peak of expansion as well. In children, no increase in more mature (CD20-CD138- and CD20-CD138+) IgA1+ plasma cells was observed at the peak of expansion. In adolescents, a small increase of the most mature (CD20-CD138+) IgA1+ plasma cells was observed, and in both adult cohorts, an increase in both intermediate and the most mature (CD20-CD138- and CD20-CD138+) IgA1+ plasma cells was observed at the peak of plasma cell expansion (adolescent cohort: *p* < 0.05, adult cohorts: *p* ≤ 0.01). When comparing all cohorts at 7 days postvaccination, a higher expansion of the most mature IgA1+ plasma cells was observed in older adults compared with children (*p* ≤ 0.01). Thus, the expansion and maturation of IgA1+ plasma cells seemed to increase with the age of the cohort. However, within the oldest cohort, no correlation was found between total IgA plasma cells, IgA1+ plasma cells, or vaccine-specific IgA cells, and age.

### 3.6. No Clear Changes in the Memory B-Cell Compartment over Time Following Vaccination 

While mature antibody-secreting plasma cells predominantly reside in bone marrow and only transiently appear in blood, memory B cells form the circulating component of immunological memory. In the steady state, memory B cells (directed against various antigens) are known to be abundant in blood of both children and adults, and undergo limited quantitative changes following antigen exposure [44,45]. Still, memory B cells with specific reactivities can be detected in the blood stream [45,46,47]. In our previous study, we showed that even minor expansions in circulating memory B cells can strongly correlate with a postvaccination increase in Bp-specific serum Ig levels [33]. Therefore, we set out to determine whether any quantitative changes can be observed at selected time points and whether the same pattern is shared by different cohorts.

**Figure 4:**
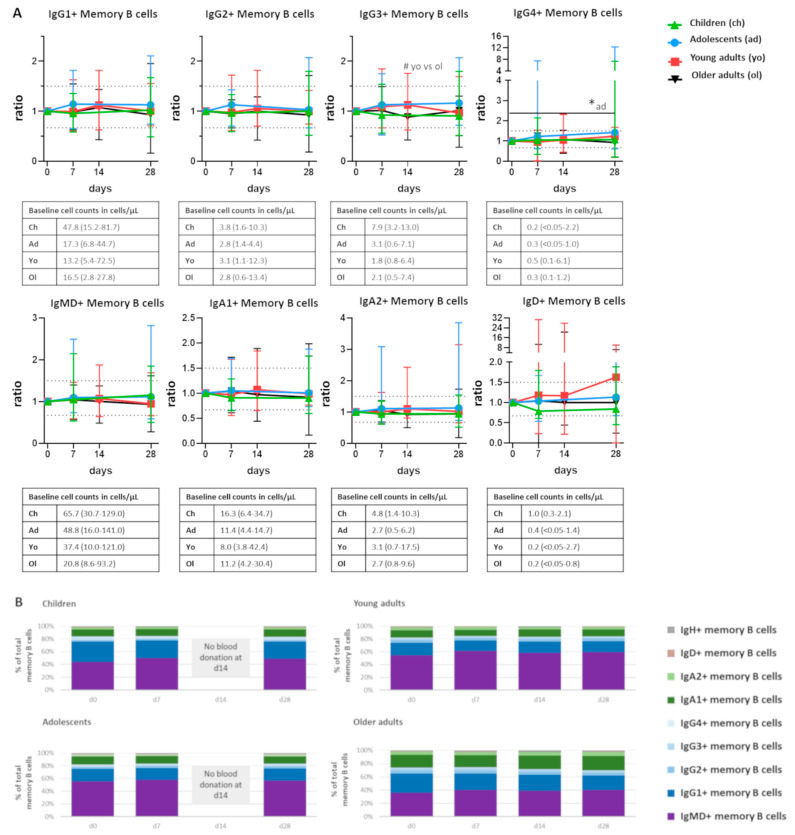
Stable distribution of memory B-cell subsets over time after vaccination. (**A**) Quantitative changes in memory B cells expressing different Ig subclasses, presented as ratio over baseline (median, min-max). Dashed lines indicate a ratio over baseline of 0.67 and 1.5. Underneath each graph, a table shows the baseline cell counts of that population in cells/µL (median, min-max). (**B**) Over-time distribution of memory B cells expressing different Ig subclasses within the total memory B-cell compartment. Median values for each population were used to construct the plots. Wilcoxon matched pair signed-rank test followed by Bonferroni correction was used to assess longitudinal differences within each cohort. Differences between cohorts were assessed using Kruskal–Wallis followed by Dunn’s test but did not yield significant differences. For pediatric cohorts, no blood samples were collected at day 14; the Mann–Whitney test followed by Bonferroni correction was used. For longitudinal changes, only significant differences compared with baseline are shown as *, *p* < 0.05. Significant differences between cohorts at the same time point are indicated as #, *p* < 0.05. d = days after vaccination.

Neither total memory B cells nor any of the major memory B-cell subsets underwent consistent quantitative changes over time following vaccination (Figure 4A). One exception was the IgG4+ memory B-cell subset in adolescents, which showed a minor but significant increase at day 28 compared with baseline levels. Moreover, at 14 days postvaccination, there was a minor but significant difference between the number of IgG3+ memory B cells between young and older adults, although there were no significant longitudinal changes within any of the groups. Within each cohort, the distribution of memory B-cell subsets was stable over the time of analysis (Figure 4B). However, upon further subdivision of memory B cells based on the expression of CD20, CD21, CD24, and CD27, two memory B-cell subsets underwent significant fluctuations over time (Supplemental Figure S3). In adolescents, there was a significant increase in IgG1+ CD20++CD21-CD24+ memory B cells at day 28 after vaccination. Moreover, in adolescents and older adults, there was a significant increase in IgG1+ CD20++CD21-CD24-CD27+ memory B cells at days 14 or 28 after vaccination compared with baseline. Therefore, it is possible that memory B cells specific to Boostrix-IPV vaccine reside within these CD20++CD21- memory B cells. Here, the use of an Ag-specific approach should lead to additional insights. No significant differences were observed between the cohorts. Although most of subsets defined within IgG1+ memory B cells were significantly more numerous in children than in both adult cohorts (data not shown), this was mainly due to higher numbers of IgG1+ memory B cells in children at baseline. Thus, except for a few minor fluctuations, no differences in the number of memory B cells were observed after Tdap booster vaccination. 

### 3.7. Good Correlation between the Increase in Plasma Cell Numbers with the Vaccine-Specific Antibody-Producing Cells 

Plasma cells are the main producers of antibodies, and, in a recall response, are mainly generated from Ag-specific memory B cells originating from a previous encounter. To support our flow cytometry-based monitoring of memory B- and plasma cell fluctuations, we determined the increase in numbers of vaccine-specific plasma and memory B cells upon vaccination via ELISpot. 

**Figure 5:**
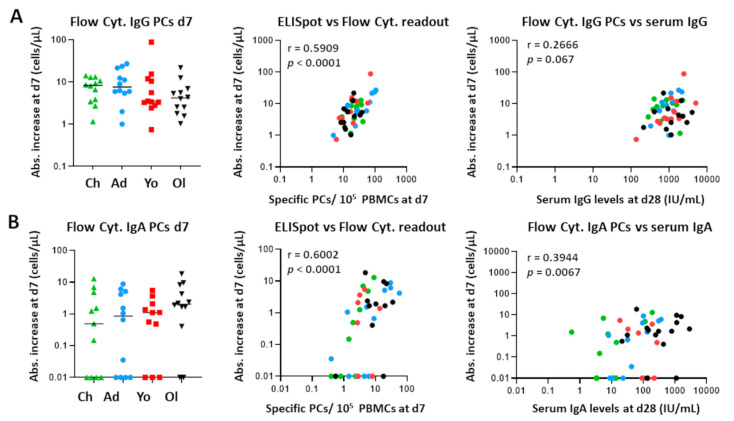
Correlation between cellular changes as measured by flow cytometry and ELISpot, and the correlation between plasma cell expansion and vaccine-specific serum Igs 28 days postvaccination. (**A**) Left panel: expansion of IgG+ plasma cells (day 7) per individual, expressed as absolute increase in cells/μL. Middle panel: correlation between the ELISpot and flow cytometry readout for IgG+ plasma cells. Right panel: correlation between the increase in plasma cells (as measured by flow cytometry) and the vaccine-specific serum IgG levels (day 28). (**B**) Left panel: expansion of IgA1+ plasma cells (day 7) per individual, expressed as absolute increase in cells/μL. Middle panel: correlation between the ELISpot and flow cytometry readout for IgA+ plasma cells. Right panel: correlation between the increase in plasma cells (as measured by flow cytometry) and the vaccine-specific serum IgA levels (day 28). Each dot represents a single donor. Of note, for visualization purposes, all absolute increases lower than 0.01 were set to 0.01. The original values were used to calculate the Spearman correlations. Flow Cyt. = flow cytometry; PCs = plasma cells; d = days after vaccination; Abs. = absolute.

Previous studies on influenza have found that Ag-specific plasma cells generated after vaccination represent up to 80% of the total plasma cell pool [30,31]. Therefore, we correlated numbers of total plasma cells (non-Ag-specific) as determined by flow cytometry to those specific for the vaccine components as determined by ELISpot analysis. In our Tdap-IPV vaccination study, the absolute increase in IgG and IgA plasma cell numbers from baseline to day 7 and the number of vaccine-specific IgG- and IgA-producing plasma cells at day 7 showed a positive correlation, indicating that the increase in total IgG and IgA plasma cells is explained by the increase in Ag-specific IgG and IgA plasma cells (IgG: r = 0.59, *p* < 0.0001; IgA: r = 0.60, *p* < 0.0001) (Figure 5). 

Next, we evaluated whether the expansion of circulating IgG+ and IgA+ plasma cells at day 7 could predict the increase in serum IgG and IgA at day 28 and year 1 postvaccination. We correlated the increase in IgG+ and IgA+ plasma cells (measured by flow cytometry) with the levels of vaccine-specific serum IgG and IgA at day 28 and year 1. Although plasma cells had no predictive value for serum IgG levels, a positive correlation was found for serum IgA levels (r = 0.3944, *p* = 0.0067). Although weaker, this correlation was still present at year 1 postvaccination (r = 0.3403, *p* = 0.0207).

For memory B cells, no correlation was observed between the ELISpot readout and flow cytometry readout (Supplemental Figure S4). Neither flow cytometry IgG nor IgA memory readout correlated with vaccine-specific serum Igs at day 28 or year 1 (data not shown). These findings indicate that for analysis of memory B cells by flow cytometry, an Ag-specific approach is required. As differences in cellular changes could be found between the different age groups, we also tested for correlations between cell expansion (ratio over baseline) and vaccine-specific serum Ig (IU/mL) within each age group (Supplemental Figure S5). No correlations between cell expansion and serum Ig levels were found. 

Thus, the expansion of total plasma cells measured by flow cytometry correlated with the expansion of Ag-specific plasma cells measured by ELISpot. In this regard, flow cytometry and ELISpot can provide complementary data. This is not observed for memory B cells.

### 3.8. Weak Positive Correlation between Plasma Cell Expansion and Vaccine-Component-Specific Ig Levels

As the response to individual vaccine components may differ, we next correlated the IgG1+ and IgA1+ plasma cell expansions at day 7 postvaccination with the levels of serum IgG and IgA directed against individual pertussis vaccine components. Additionally, we tested whether the degree of maturation correlated with vaccine-component-specific IgG or IgA levels. Within the IgG1+ plasma cells, the strongest correlations (with a correlation coefficient between 0.3–0.5) were found between PT- or Prn-specific IgG levels and total IgG1+ plasma cells or CD20-CD138- IgG1+ plasma cells (Supplemental Table S5 (IgG). No correlations were observed between IgG1+ memory B-cell expansion and vaccine-component-specific serum IgG. Correlations between IgA1+ plasma cells and serum IgAs against individual pertussis components were higher and more frequent (ranging between 0.3–0.6, Supplemental Table S6). Most correlations were found between FHA-specific serum IgAs and the increase in total IgA1+ plasma cells, CD20-CD138- IgA1+ plasma cells, and CD20-CD138+ IgA1+ plasma cells. Considerably fewer correlations were found between PT- and Prn-specific serum IgAs and the increase in IgA1+ plasma cells. Interestingly, a positive correlation was found between the maximum expansion of IgA1+ memory B cells and the FHA-specific serum IgAs at year 1 postvaccination (ratio over baseline).

### 3.9. More Prominent Cellular Responses in Participants Primed with wP Vaccine

Due to the change in the National Immunization Program on 1 January 2005, all children and 5 out of 12 adolescents were primed with an aP vaccine, while 7 adolescents and all young adults received the Dutch wP vaccine in childhood (presumably, older adults were vaccinated with wP or had received no pertussis vaccination during childhood). It has been previously shown that the primary vaccine can impact both T-cell and antibody responses [16,18,22,23,24]. As antibodies are the product of plasma cells, we studied whether B-cell and plasma cell responses are also influenced by the primary vaccine. To avoid the effect of age, we first compared both subgroups of adolescents.

**Figure 6:**
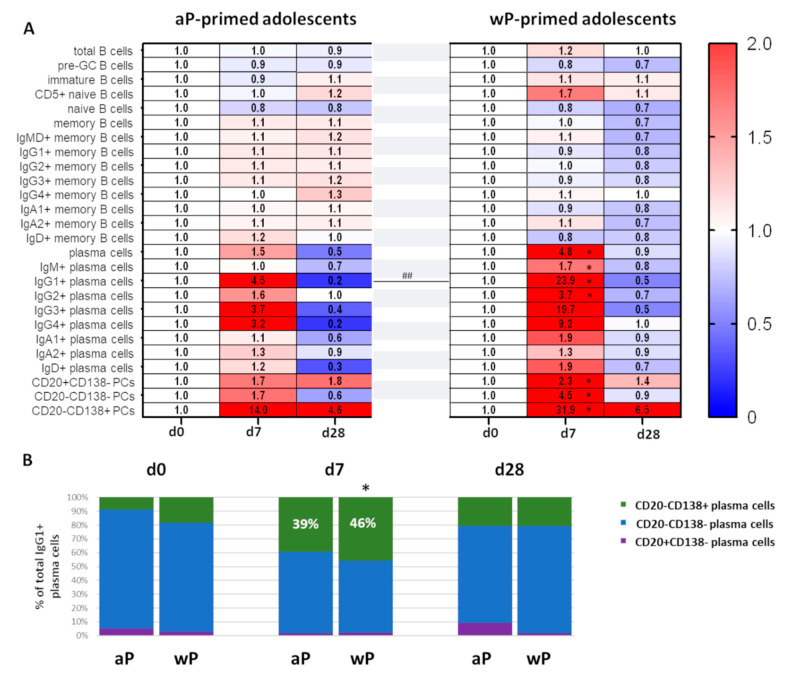
IgG1+ plasma cell expansion and maturation are more prominent in age-matched participants after wP priming. (**A**) Heatmap showing over-time changes in memory B-cell and plasma cell subsets in aP-primed (*n* = 5) or wP-primed (*n* = 7) adolescents (median values). (**B**) Over-time distribution of IgG1+ plasma cells representing different maturation stages within total IgG1+ plasma cells. Median values for each population were used to construct the plots. Wilcoxon matched pair signed-rank test followed by Bonferroni correction was used to assess longitudinal differences in percentage of CD20-CD138+ cells in total IgG1+ plasma cells within each cohort. Differences in the percentage CD20-CD138+ cells in total IgG1+ plasma cells between cohorts were assessed using Kruskal–Wallis followed by Dunn’s test but did not yield significant differences. For longitudinal changes, only significant differences compared to baseline are shown. Significant longitudinal differences within a cohort are indicated as *, *p* < 0.05. Significant differences between cohorts at the same time point are indicated as ##, *p* ≤ 0.01. d = days after vaccination; aP = acellular pertussis vaccine; wP = whole-cell pertussis vaccine; pre-GC = pre-Germinal Center.

Over-time changes in numbers of total, naive, and memory B cells were minor and comparable between wP- and aP-primed adolescents (Figure 6A). In contrast, changes in plasma cell numbers were much more prominent in adolescents who were primed with the Dutch wP vaccine vs. aP-primed adolescents (ratio to baseline: 4.8 vs. 1.5 at day 7 in total plasma cell numbers). From all plasma cells, the differences were the clearest for IgG1+ (ratio: 23.9 vs. 4.5 at day 7), IgG3+ (ratio: 19.7 vs. 3.7 at day 7), and IgG4+ (ratio: 9.2 vs. 3.2 at day 7) plasma cells. Only the difference in IgG1+ plasma cell expansion reached statistical significance (*p* ≤ 0.01), possibly due to the low number of participants in both groups. Moreover, plasma cell maturation at the peak of expansion was more prominent in wP-primed adolescents in whom the most mature CD20-CD138+ plasma cells constituted 46% of IgG1+ plasma cells in contrast to 39% in aP-primed adolescents (Figure 6A,B). Thus, the type of primary vaccination background seems to influence the plasma cell response to later booster vaccinations. In our study, the plasma cell response was stronger and more diverse in wP-primed adolescents.

To exclude that differences observed in the adolescent cohort were caused by the different sex distribution between aP- and wP-primed individuals, we assessed the impact of sex on the cell expansion in the young adult cohort and extrapolated this to the adolescent cohort. The young adult cohort was well sex-balanced and had a homogenous priming background. We evaluated the expansion of total B cells, plasma cells, IgG1–3+ plasma cells, and IgA1+ plasma cells in males and females. No significant differences were observed between male and female responses (Supplemental Figure S6). Therefore, we concluded that the sex imbalance in the adolescent cohort did not influence the plasma cell expansion as found in this study.

The adolescent cohort only consisted of 12 individuals, which is rather small for statistical analysis. Therefore, although we found some age-dependent differences in cellular responses, we grouped all aP-primed individuals (children and adolescents) and all wP-primed individuals (adolescents and young adults; not the older adults, because of their uncertain vaccination background status) to increase the size of the aP- vs. wP-primed study cohorts (Supplemental Figure S7A). In this comparison, expansions of total and IgG1+ plasma cells were again more pronounced in wP-primed participants. Differences in other plasma cell subsets did not reach statistical significance. Finally, based on ratio to baseline, the (total) plasma cell maturation was more prominent in wP-primed individuals compared with aP-primed individuals, and differed significantly for intermediate mature plasma cells. The percentage of the most mature cells in the IgG1+ plasma cell population did not significantly differ between the two groups (Supplemental Figure S7).

In summary, we showed that, irrespective of the age of vaccinated individuals, the most prominent cellular changes occurred in the numbers of circulating plasma cells. Despite some age-related differences, the expansion and maturation of IgG1+ plasma cells at day 7 postvaccination are a shared phenomenon. This expansion of plasma cells measured by flow cytometry was complementary to the increase of vaccine-specific plasma cell numbers determined by ELISpot. Positive correlations between plasma cell expansion and postvaccination Ag-specific serum Ig levels were observed, mainly when correlating with the individual Bp components (Bp: FHA, Prn, and PT). Finally, plasma cell responses were stronger in individuals who were wP-primed.

## 4. Discussion

In this study, we applied high-dimensional flow cytometry to investigate changes in B cells in individuals of different ages and primary vaccination backgrounds upon administration of an aP booster vaccine and correlated these findings with vaccine-specific Ig levels in serum. In all age groups, expansion and maturation of plasma cells 7 days postvaccination was the most prominent cellular change. Although in children the expansion of plasma cells was less prominent than in adults (ratio to baseline), they had more plasma cells at peak levels due to their initially high plasma cell numbers. Furthermore, total and IgG1+ plasma cell responses were stronger in individuals primed with the Dutch wP vaccine than in individuals who were primed with aP vaccines. No consistent over-time memory B-cell fluctuations were observed. No strong correlation between plasma cell expansion or memory B-cell expansion with vaccine-specific serum Ig levels was observed, yet the absolute increase in IgA plasma cells at day 7 correlated weakly with the IgA serum levels at day 28 (IU/mL). Furthermore, weak positive correlations were observed between the expansion of IgG1+ and IgA1+ plasma cells and FHA-, Prn-, and PT-specific serum IgG or IgA levels postvaccination. Although serology provides insight into Ag-specific Ig levels and function, analysis of circulating immune cells may result in a deeper understanding of the processes induced by the vaccine and the cellular changes preceding Ig production. Our study points at plasma cells as a potential cellular marker of an immune response and contributes to a better understanding of the immune responses (to booster vaccinations) between different age groups and different primary vaccination backgrounds.

To ensure objective data analysis, we used the automated gating and identification (AGI) tool in the Infinicyt software. This tool was shown to reduce intra- and inter-operator variability and increase reproducibility of the analysis [40,48,49,50]. This is especially important for studies with big data from multiple samples, which cannot be analyzed by a single operator within a reasonable timeframe. Irrespective of the new analysis strategy, these data corroborated major findings from our previous study, where data were subjected to manual analysis [33]. This automated analysis approach, in combination with the standardized EuroFlow sample processing and acquisition procedures, allows for identification of fluctuations in small populations of cells such as different plasma cell maturation stages.

Levels of Ag-specific serum Igs are routinely used as readout for vaccine efficacy. In many cases, a rise in Ag-specific IgG levels is associated with response to vaccination, and for several vaccines—e.g., against rotavirus—an increase in IgA levels has been indicated as a correlate of protection [23,45,51,52,53]. As Igs are the product of terminally differentiated B cells (plasma cells), the B-cell compartment may harbor new correlates or biomarkers of ongoing immune responses. Indeed, we found that expansion and maturation of circulating plasma cells 7 days after booster vaccination was the most prominent cellular change. The generation of mainly IgG1+ plasma cells is in line with previous serology-based studies, where within Bp-specific serum IgGs mostly IgG1 antibodies were found, with minor contribution of IgG2, -3, and -4 [51,54]. The positive correlation between the numbers of total plasma cells with the vaccine-specific plasma cell numbers supports the assumption that most of the plasma cells at the peak of expansion are vaccine-specific.

The IgA response, observed mostly in the adult cohorts, is likely a result of immunological memory generated by previous (subclinical) infection of the respiratory tract, where a mucosal response against Bp was launched. As Bp circulates within the population, causing outbreaks every 2–5 years, the adult cohorts have likely encountered Bp multiple times during life, which explains the more prominent IgA1+ plasma cell response in these groups [55,56,57,58]. In contrast, the expansion of IgG4+ plasma cells was mostly seen in the pediatric cohorts, which may be explained by the predominant aP priming in these cohorts; this has been shown to induce a more Th2-related response as well as increased vaccine-specific serum IgG4 [23,24].

In addition to the expansion of IgG1+ and IgA1+ plasma cells in adults, and the IgG1+ and IgG4+ response in the pediatric cohorts, which are in line with previous (cellular and serology-based) studies, we also observed a prominent increase in IgG3+ plasma cells in all cohorts [23,33,51,54]. A potential explanation for this phenomenon might be that, in addition to the memory B cells, there are naive B cells that recognize the antigens and undergo first-step IgG3 class switching and affinity maturation [59].

The difference in plasma cell—and thus, antibody—production can have consequences for the type and efficacy of the launched immune response. IgG1 and IgG3 antibodies have stronger opsonizing capacities compared with IgG4 antibodies [60]. The mixed IgG1-IgG3-IgG4 response observed in the mostly aP-primed pediatric cohorts may lead to competition for Bp antigens in future encounters, possibly leading to less efficient bacterial clearance compared with the IgG1-IgG3 (and IgA1) response observed in the adult cohorts [59,61]. Lastly, the prominent contribution of IgA1+ plasma cells to responses observed in the adult cohorts, which is likely an indicator of previous pertussis encounters, may imply existence of effective mucosal defense mechanisms, and more efficient protection against bacterial translocation in IgA-producing individuals. Comparison of repertoires and reactivities of IgA in mucosa and in circulation could provide better insights into this phenomenon and value of IgA as a biomarker of protection.

Maturation of plasma cells (total and IgG1+) was observed irrespective of age and priming background. The clear expansion and maturation of total and IgG1+ plasma cells are in line with our previous findings and may be explained by the prolonged retention of newly generated plasma cells in the periphery as well as the competition for bone marrow niches with pre-existing long-lived plasma cells [33,62].

In this study, several differences between the aP- and wP-primed cohorts were observed. Although the sizes of the age-matched adolescent cohorts were too limited to reach statistically significant conclusions, major observations were confirmed by analysis of all individuals with known primary vaccination background. Remarkably, this difference based on primary vaccination background was not observed in the overarching part of the BERT study, where the Bp-specific Ig responses of 85 Dutch and Finnish adolescents pre- and postvaccination were evaluated [29].

The formulation of aP and wP vaccines differs with regards to number of antigens and the total antigenic load, with wP vaccines containing the broad variety of pertussis antigens and aP vaccines containing high concentrations of a restricted number of antigens. In consequence, wP priming is likely to trigger a more diverse antibody response. Since consecutive boosters lead to a more specific, but also more restricted response, this initial broad priming can be beneficial in case of encountering future (mutated) bacterial strains [10,12,13]. Interestingly, in this study, we showed that compared to aP-primed individuals, individuals primed with the Dutch wP vaccine have a stronger response upon aP booster vaccination. It would be of interest to visualize potential differences in breadth of an immune response against Bp antigens. Moreover, since the initial type of priming vaccine seems to imprint future responses to given antigens, it should be carefully considered in the design of future vaccines and vaccination strategies. This may also hold true for diseases other than pertussis, such as COVID-19.

To identify unique and shared patterns between groups, we primarily focused on normalized data (represented as the ratio to baseline). However, we also showed that, in line with published studies, children had overall higher leukocyte and B-cell counts compared with (older) adults [32,41]. Specifically, the cell count of naive B and T cells—and thus, the available naive repertoire—is substantially higher in youth [32,48]. Therefore, despite a lower increase in cells expressed as ratio to baseline, children and adolescents may still produce a stronger and more diverse immune responses than adults.

In this study, memory B-cell fluctuations were limited. As the frequencies of Ag-specific memory B cells are low, as demonstrated by previous studies using ELISpot assays, an increase in only these Ag-specific memory B cells may not have an impact on the total memory B-cell population [36,63]. Indeed, in this study, no correlation was found between the memory B-cell fluctuations measured by flow cytometry and the vaccine-specific memory B cells by ELISpot. However, we observed an increase in CD20++CD21- IgG1+ memory B cells in older adults and adolescents at days 14 and 28 after vaccination, respectively. Interestingly, several studies reported an increased percentage of Ag-specific CD27+CD21-/dim B cells 14 days after influenza vaccination [64,65]. There is no consensus about the exact function of these CD21-/dim B cells, but it has been suggested that CD21-/dim B cells are exhausted cells or, as described in autoimmunity and chronic infection, are anergic [66,67]. In this context, it is not unlikely that cells that have responded to an antigen multiple times would acquire this phenotype. However, Lau and colleagues suggest that CD21-/dim B cells are primed for plasma cell differentiation [64]. Ag-specific flow cytometry studies should give insight into the exact function of this B-cell subset.

No correlation was found between expansion of memory B cells and Ag-specific serum IgG levels at day 28. Previously, we observed a clear correlation between the expansion of IgG1+ memory B cells and the vaccine-specific IgG levels at day 21 in a cohort of 10 healthy adults [33]. Moreover, we found that although in the majority of participants memory B cells showed maximum expansion at 14 days after vaccination, the expansion of memory B cells was not as synchronized in time as the plasma cell expansion, implying that, in some participants, we may not have sampled at the most optimal timepoint [33], especially in the children and adolescent cohorts, where the sampling times were limited to days 0, 7, and 28. This difference in timing of memory B-cell responses might be related to the immune status of each individual at baseline and makes the use of memory B cells as correlates of protection more difficult.

Neither aP nor wP vaccination yield a response that fully mimics natural infection; especially, the IgA response seems to be limited upon vaccination and mostly relies upon previous encounters with Bp. To overcome this limitation, multiple novel pertussis vaccines and alternative delivery routes are being developed, such as nasal delivery of a vaccine or the use of life-attenuated Bp strains (BPZE1) [68,69,70]. It would be of interest to evaluate how (cellular) immune responses induced by these vaccine candidates compare with cellular kinetics induced by intramuscular aP and wP vaccines, as well as (controlled) human infection. Such comparison between aP booster vaccination and (controlled) human infection is currently ongoing within the IMI-2 PERISCOPE program [71,72]. These studies will create a solid basis for evaluation of novel vaccination approaches.

## 5. Conclusions

Analysis of circulating immune cells results in a deeper understanding of the processes induced by vaccination and the cellular changes preceding Ig production. Irrespective of the age of vaccinated individuals, the most prominent cellular changes occurred in the numbers of circulating plasma cells. The expansion and maturation of IgG1+ plasma cells at day 7 postvaccination were a shared phenomenon. Plasma cell expansion, as determined by flow cytometry, was complementary to the increase of vaccine-specific plasma cell numbers as determined by ELISpot. Positive correlations between plasma cell expansion and postvaccination Ag-specific serum Ig levels were observed, mainly when correlating with the individual Bp components. Finally, plasma cell responses were stronger in individuals who were wP-primed. Thus, our study contributes to a better understanding of the immune responses (to booster vaccinations) between different age groups and different primary vaccination backgrounds.

## Figures and Tables

**Table 1:** Baseline distribution of normal B-cell and plasma cell subsets (cells/µL) in each age cohort. Ages of the cohorts of which reference values were used: children 5–9 y/o, adolescents 10–17 y/o, young adults 18–39 y/o, older adults 60–79 y/o. The reference values as indicated in this table were selected from the study performed by Blanco et al. [32]. In the before-mentioned study, reference values for B-cell subsets are provided for all age ranges (from cord blood until older adults >80 y/o). When referring to the reference values indicated here, please refer to the original source data.

Cohort	Children (ch)			Adolescents (ad)			Young Adults (yo)			Older Adults (ol)			Statistical Differences Between Cohorts				
Age	7–10 y/o			11–15 y/o			20–34 y/o			60–70 y/o			No significant differences found within the other combination (yo vs. ol)				
Priming background	aP			Mixed aP and wP			wP			No vaccination history available (presumably wP-primed or not vaccinated)							
N=	11 ^†^			12			11 ^†^			12							
	median	min.	max.	median	min.	max.	median	min.	max.	median	min.	max.	ch vs. yo	ch vs. ol	ch vs. ad	ad vs. yo	ad vs. ol
total B-cell reference cell counts cell counts	451 496.0	157 346.0	725 966.0	360 430.0	174 133.0	630 667.0	220 279.0	41 141.0	470 481.0	173 244.0	36 53.5	384 396.0	**	***	ns	ns	*
pre-GC B-cell reference cell counts cell counts	391.0	NI 173.0	731.0	326.5	NI 96.3	529.0	194.0	NI 53.2	342.0	195.0	NI 31.5	273.0	**	**	ns	*	**
immature B-cell reference cell counts cell counts	41 26.6	12 9.5	84 70.4	37 20.8	11 1.6	111 43.7	5.6 5.9	0.25 1.1	24 19.8	6.1 4.7	0.69 0.6	36 9.2	**	****	ns	*	**
CD5+ naive B-cell reference cell counts cell counts	89.7	NI 25.0	248.0	49.8	NI 5.2	166.0	30.9	NI 7.8	152.0	15.3	NI 2.4	47.0	*	***	ns	ns	*
naive B-cell reference cell counts cell counts	265 260.0	68 124.0	505 483.0	189 238.5	75 89.4	401 335.0	111 160.0	13 44.0	288 186.0	109 150.0	20 24.9	280 250.0	**	*	ns	ns	*
memory B-cell reference cell counts cell counts	123 160.0	64 76.1	282 252.0	68 96.2	31 33.6	1 60 193.0	91 85.7	23 31.3	221 282.0	56 63.8	13 21.1	128 171.0	ns	**	ns	ns	ns
**IgMD+ memory cells** **reference cell counts** **cell counts**	54 65.7	23 30.7	147 129.0	29 48.8	17 16.0	78 141.0	38 37.4	7.9 10.0	122 121.0	27 20.8	7.4 8.6	72 93.2	*	****	ns	ns	ns
**IgG1+ memory cells** **reference cell counts** **cell counts**	30 47.8	12 15.2	86 81.7	18 17.3	7 6.8	42 44.7	18 13.2	3.2 5.4	40 72.5	9.1 16.5	1.3 2.8	22 27.8	*	****	ns	ns	**
**IgG2+ memory cells** **reference cell counts** **cell counts**	4.4 3.8	0.7 1.6	15 10.3	3.0 2.8	0.7 1.4	10 4.4	5.9 3.1	1.6 1.1	30 12.3	3.6 2.8	1.0 0.6	11 13.4	*	***	ns	ns	ns
**IgG3+ memory cells** **reference cell counts** **cell counts**	7.4 7.9	2.4 3.2	16 13.0	3.0 3.1	1.1 0.6	8.3 7.1	3.0 1.8	0.5 0.8	8.4 6.4	2.3 2.1	0.4 0.5	8.1 7.4	*	**	ns	ns	ns
**IgG4+ memory cells** **reference cell counts** **cell counts**	0.4 0.2	<0.01 <0.05	2.0 2.2	0.2 0.3	<0.01 <0.05	2.9 1.0	0.4 0.5	<0.01 0.1	2.4 6.1	0.4 0.3	<0.01 0.1	2.1 1.2	ns	***	ns	ns	ns
**IgA1+ memory cells** **reference cell counts** **cell counts**	12 16.3	4.5 6.4	24 34.7	9.0 11.4	2.9 4.4	21 14.7	11 8.0	2.1 3.8	43 42.4	6.2 11.2	2.2 4.2	22 30.4	ns	ns	ns	ns	ns
**IgA2+ memory cells** **reference cell counts** **cell counts**	3.2 4.8	1.0 1.4	13 10.3	2.7 2.7	0.8 0.5	5.9 6.2	4.1 3.1	1.2 0.7	18 17.5	3.4 2.7	0.7 0.8	9.0 9.6	ns	**	ns	ns	ns
**IgD+ memory cells** **reference cell counts** **cell counts**	1.1 1.0	<0.01 0.3	2.9 2.1	0.3 0.4	<0.01 <0.05	1.7 1.4	0.2 0.2	<0.01 <0.05	2.4 2.7	0.01 0.2	<0.01 <0.05	1.2 0.8	*	**	ns	ns	ns
**IgH- memory cells** **reference cell counts** **cell counts**	2.2	NI 0.6	4.4	1.5	NI 0.4	2.7	1.2	NI 0.5	1.8	0.9	NI 0.3	3.0	ns	ns	ns	ns	ns
**total plasma cells** **reference cell counts** **cell counts**	13 8.5	3.5 3.8	45 12.9	8.5 3.4	1.3 1.6	27 13.1	4.4 2.6	1.1 1.2	25 8.5	1.2 1.6	0.3 0.2	7.1 6.1	ns	*	ns	ns	ns
**IgM+ plasma cells** **reference cell counts** **cell counts**	1.4 1.3	0.6 0.3	14 2.9	0.8 0.6	0.2 0.4	5.7 2.0	0.4 0.4	0.05 0.1	4.7 2.8	0.1 0.1	0.01 <0.05	0.8 0.8	ns	ns	ns	ns	ns
**IgG1+ plasma cells** **reference cell counts** **cell counts**	1.9 1.5	0.1 0.2	7.7 2.7	1.1 0.4	0.1 0.1	4.8 5.6	0.4 0.4	0.05 0.1	4.4 1.3	0.1 0.2	0.01 0.1	0.6 0.6	**	**	ns	ns	ns
**IgG2+ plasma cells** **reference cell counts** **cell counts**	0.7 0.8	0.07 0.2	2.3 1.1	0.5 0.3	0.08 0.1	0.8 1.5	0.2 0.2	<0.01 <0.05	2.6 0.6	0.09 0.1	<0.01 <0.05	1.6 1.2	ns	ns	ns	ns	ns
**IgG3+ plasma cells** **reference cell counts** **cell counts**	0.2 0.2	<0.01 <0.05	1.3 0.5	0.08 <0.05	<0.01 <0.05	0.4 0.9	0.03 <0.05	<0.01 <0.05	0.3 0.1	<0.01 <0.05	<0.01 <0.05	0.2 0.1	*	**	ns	ns	ns
**IgG4+ plasma cells** **reference cell counts** **cell counts**	0.02 <0.05	<0.01 <0.05	0.2 0.2	<0.01 <0.05	<0.01 <0.05	0.2 0.2	<0.01 <0.05	<0.01 <0.05	0.4 0.7	<0.01 <0.05	<0.01 <0.05	0.1 0.1	ns	ns	ns	ns	ns
**IgA1+ plasma cells** **reference cell counts** **cell counts**	4.4 3.4	0.6 0.6	16 5.6	3.1 1.3	0.5 0.4	14 4.1	1.7 1.1	0.3 0.4	6.9 3.3	0.4 0.7	0.04 0.1	3.3 3.0	ns	ns	ns	ns	ns
**IgA2+ plasma cells** **reference cell counts** **cell counts**	1.5 1.0	0.3 0.3	3.5 2.7	1.0 2.7	0.3 0.5	3.6 6.2	0.7 0.3	0.2 0.1	4.2 1.1	0.3 0.2	0.06 0.1	1.2 1.2	ns	ns	ns	ns	ns
**IgD+ plasma cells** **reference cell counts** **cell counts**	0.04 0.1	<0.01 <0.05	0.8 0.6	<0.01 <0.05	<0.01 <0.05	2.0 0.1	<0.01 <0.05	<0.01 <0.05	1.1 0.1	<0.01 <0.05	<0.01 <0.05	0.2 <0.05	**	**	*	ns	ns

^†^ in children and young adults, for one included donor no baseline B-cell data was available. NI = not indicated. * *p* < 0.05; ** *p* ≤ 0.01; *** *p* ≤ 0.001; **** *p* ≤ 0.0001.

## Data Availability

The data presented in this study are available on request from the corresponding author.

## Supplementary Materials

The following supporting information can be downloaded at: https://www.mdpi.com/article/10.3390/vaccines10020136/s1, Supplemental Table S1. Complete overview of the inclusion and exclusion criteria for this study. Supplemental Table S2. Composition of the EuroFlow B-cell panel and technical information on the reagents for the IMI-2 PERISCOPE BERT study. Supplemental Table S3. Phenotypic descriptions used to define B-cell subsets stained with the EuroFlow B-cell panel by manual analysis. Supplemental Table S4. Baseline distribution of leukocytes, lymphocytes, T cells, and NK cells in donor groups. Supplemental Table S5. Spearman Ranking Correlation between IgG1+ plasma cell and memory B-cell kinetics and vaccine-component-specific serum IgG. Supplemental Table S6. Spearman Ranking Correlation between IgA1+ plasma cell and IgA memory B-cell kinetics and vaccine-component-specific serum IgA. Supplemental Figure S1. No clear over-time postvaccination changes in major populations in any of the donor groups. Supplemental Figure S2. Over-time maturation of total plasma cells. Supplemental Figure S3. No significant changes in IgG1+ memory B-cell subsets upon vaccination. Supplemental Figure S4. Correlation between cellular changes as measured by flow cytometry and ELISpot. Supplemental Figure S5. Correlation between cellular changes and the vaccine-specific serum IgG level postvaccination as determined by Spearman’s Ranking Correlation per age cohort. Supplemental Figure S6. Impact of sex on cellular responses after vaccination in the young adult cohort (all wP-primed). Supplemental Figure S7. IgG1+ and total plasma cell expansion is more prominent in non-age-matched donors after wP priming.
