# Supplementary material for: Age and Primary Vaccination Background Influence the Plasma Cell Response to Pertussis Booster Vaccination
Source: Vaccines (Basel). 2022 Jan 18;10(2):136. doi: 10.3390/vaccines10020136 (PMC8878388; doi:10.3390/vaccines10020136)
Supplement: Supplementary file 1 [file Supplemental Table S2. Diks et al. B-cell kinetics after aP vaccination_proof prints.pdf › Supplemental Table S2. Diks et al. B-cell kinetics after aP vaccination_proof prints.pdf]

Table S2: Composition of the EuroFlow B-cell panel and technical information on the reagents for the IMI-2 PERISCOPE BERT study.

**Marker**	CD27	IgM	CD62L	CD24	CD21	CD19	IgD	IgH-isotype panel – Subclasses (CYT-IGS-1)	CD20	CD5	CD138	IgD	CD45	CD38
**Fluorochrome**	BV421	BV510	BV605	BV650	BV711	BV786	FITC	-	PE CF594	PE Cy7	PE Cy7	APC	Alexa Fluor 700	APC H7
**Manufacturer**	BD	Biolegend	Biolegend	BD	BD	BD	Biolegend	Cytognos	BD	BD	Biolegend	BD	BD	BD
**Clone**	M-T271	MHM-88	DREG-56	ML5	B-Ly4	SJ25C1	IA6-2	n/a	2H7	L17F12	MI15	IA6-2	HI30	HB7
**Volume (µl)**	2	2	5	5	5	4	1.25	25	0.25	6	5	4	10	3
**Membrane stain**	All markers are used for membrane stain.													
**Intracellular stain**	IgM BV510, IgD FITC, IgH-isotype panel- subclasses (CYT-IGS-1), IgD APC are used for additional intracellular stain.													
