# Supplementary material for: Age and Primary Vaccination Background Influence the Plasma Cell Response to Pertussis Booster Vaccination
Source: Vaccines (Basel). 2022 Jan 18;10(2):136. doi: 10.3390/vaccines10020136 (PMC8878388; doi:10.3390/vaccines10020136)
Supplement: Supplementary file 1 [file vaccines-10-00136-s001.zip › Supplemental Figures. Diks et al. B-cell kinetics after aP vaccination_proof prints.pdf]

Supplementary Material

Supplemental Tables

Table S1. Complete overview of the inclusion and exclusion criteria for this study.

Table S2. Composition of the EuroFlow B-cell panel and technical information on the reagents for the IMI-2 PERISCOPE BERT study.

Table S3. Phenotypic descriptions used to define B-cell subsets stained with the EuroFlow B-cell panel by manual analysis.

Table S4. Baseline distribution of leukocytes, lymphocytes, T cells and NK cells in donor groups.

Table S5. Spearman Ranking Correlation between IgG1+ plasma cell and memory B-cell kinetics and vaccine component-specific serum IgG.

Table S6. Spearman Ranking Correlation between IgA1+ plasma cell and IgA memory B-cell kinetics and vaccine component-specific serum IgA.

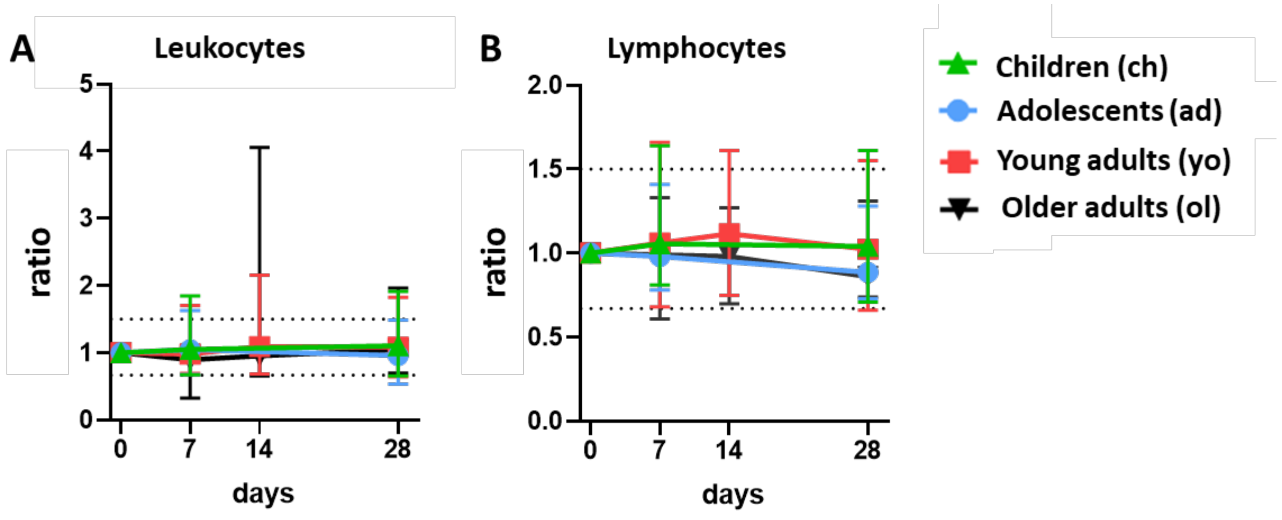

Baseline cell counts in cells/µL	
Ch	5795 (3397-14263)
Ad	6148 (3296-7837)
Yo	5054 (3224-6501)
Ol	5234 (3373-7547)

Baseline cell counts in cells/µL	
Ch	2551 (2128-3902)
Ad	2389 (1077-3312)
Yo	2017 (1699-3057)
Ol	2003 (1150-3080)

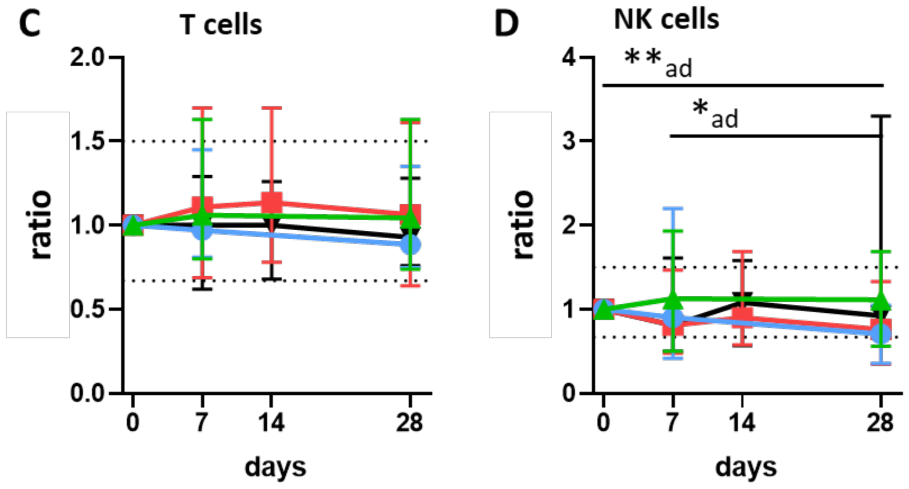

Baseline cell counts in cells/µL	
Ch	1806 (1132-3308)
Ad	1674 (814-2466)
Yo	1481 (1204-2405)
Ol	1481 (794-2312)

Baseline cell counts in cells/µL	
Ch	210 (123-508)
Ad	268 (65-456)
Yo	214 (115-713)
Ol	282 (87-547)

Figure S1. No clear over-time post-vaccination changes in major populations in any of the donor groups. The post-vaccination fluctuations of (**A**) leukocytes, (**B**) lymphocytes, (**C**) T cells, and (**D**) NK cells, presented as ratio over baseline (median, min-max). Dashed lines

indicate a ratio of 0.67 and 1.5 compared to baseline. Underneath each graph, the baseline cell counts per cohort are presented in cells/μL (median, min-max). To assess longitudinal changes within each cohort, Wilcoxon matched pair signed-rank test followed by Bonferroni correction was used. To test differences between cohorts at one timepoint, Kruskal-Wallis followed by Dunn's test was used, with exception of the comparison at day 14. At day 14, only blood samples from the adult cohorts were collected. Here, the Mann-Whitney test followed by Bonferroni correction was used. For longitudinal changes, only significant differences compared to baseline are shown. *, $p < 0.05$; **, $p \leq 0.01$.

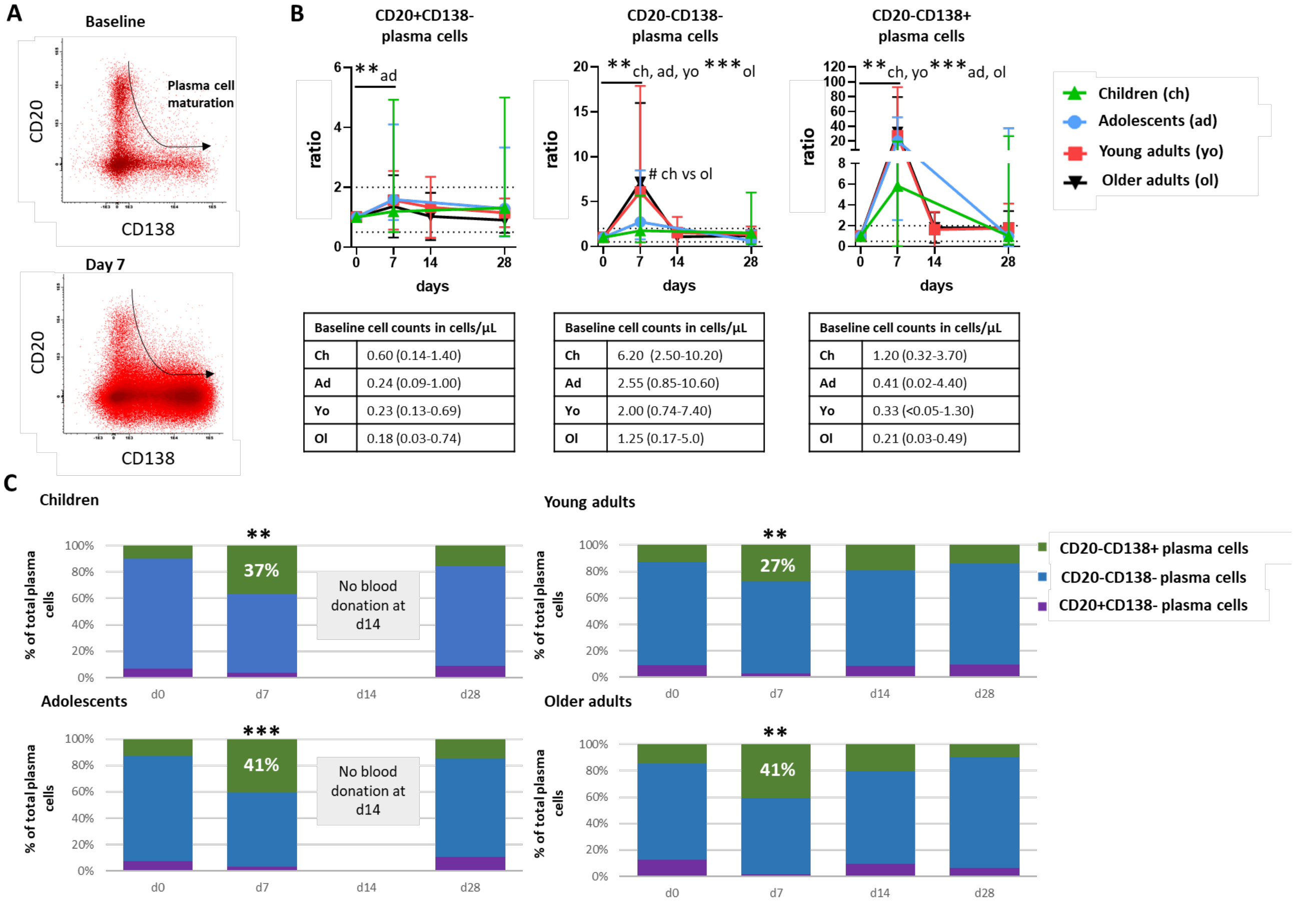

Baseline cell counts in cells/µL	
Ch	0.60 (0.14-1.40)
Ad	0.24 (0.09-1.00)
Yo	0.23 (0.13-0.69)
Ol	0.18 (0.03-0.74)

Baseline cell counts in cells/µL	
Ch	6.20 (2.50-10.20)
Ad	2.55 (0.85-10.60)
Yo	2.00 (0.74-7.40)
Ol	1.25 (0.17-5.0)

Baseline cell counts in cells/µL	
Ch	1.20 (0.32-3.70)
Ad	0.41 (0.02-4.40)
Yo	0.33 (<0.05-1.30)
Ol	0.21 (0.03-0.49)

Figure S2. Over time maturation of total plasma cells. (A) Representative plots showing the phenotypical changes during plasma cell maturation. Each dot represents an individual cell. The arrow indicates the direction of changes during maturation. **(B)** Over-time quantitative changes in plasma cells belonging to different maturation stages, presented as ratio over baseline (median, min-max). Dashed lines indicate a ratio of 0.5 and 2.0 compared to baseline. Underneath each graph, the baseline cell counts per cohort are indicated in cells/µL (median, min-max). **(C)** Over-time distribution of plasma cells representing different maturation stages within total plasma cells. Median values for each population were used to construct the plots. Wilcoxon matched pair signed-ranked test followed by Bonferroni correction was used to assess longitudinal differences in percentage of CD20-CD138+ cells in total plasma cells within each cohort. Differences in the percentage CD20-CD138+ cells in total plasma cells between cohorts were assessed using Kruskal-Wallis followed by Dunn's test, but did not yield significant differences. At day 14, only blood samples from the adult cohorts were

collected. Here, the Mann-Whitney test followed by Bonferroni correction was used. For longitudinal changes, only significant differences compared to baseline are shown. Significant longitudinal differences within a cohort as indicated with **, $p \leq 0.01$; ***, $p \leq 0.001$. Significant differences between cohorts at the same time point are indicated with #, $p < 0.05$.

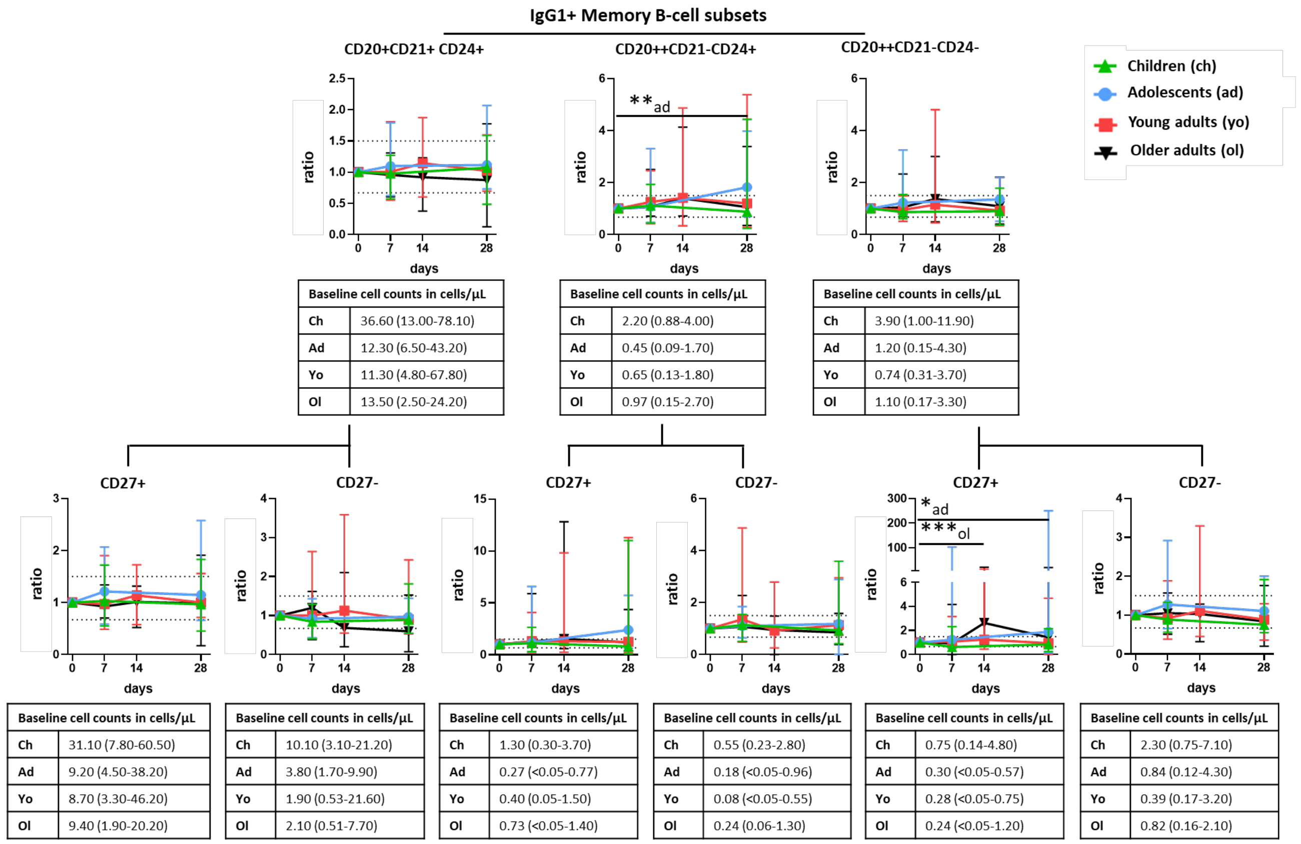

CD20+CD21+ CD24+

Baseline cell counts in cells/µL	
Ch	36.60 (13.00-78.10)
Ad	12.30 (6.50-43.20)
Yo	11.30 (4.80-67.80)
Ol	13.50 (2.50-24.20)

CD20++CD21-CD24+

Baseline cell counts in cells/µL	
Ch	2.20 (0.88-4.00)
Ad	0.45 (0.09-1.70)
Yo	0.65 (0.13-1.80)
Ol	0.97 (0.15-2.70)

CD20++CD21-CD24-

Baseline cell counts in cells/µL	
Ch	3.90 (1.00-11.90)
Ad	1.20 (0.15-4.30)
Yo	0.74 (0.31-3.70)
Ol	1.10 (0.17-3.30)

CD20+CD21+ CD24+ CD27+

Baseline cell counts in cells/µL	
Ch	31.10 (7.80-60.50)
Ad	9.20 (4.50-38.20)
Yo	8.70 (3.30-46.20)
Ol	9.40 (1.90-20.20)

CD20+CD21+ CD24+ CD27-

Baseline cell counts in cells/µL	
Ch	10.10 (3.10-21.20)
Ad	3.80 (1.70-9.90)
Yo	1.90 (0.53-21.60)
Ol	2.10 (0.51-7.70)

CD20++CD21-CD24+ CD27+

Baseline cell counts in cells/µL	
Ch	1.30 (0.30-3.70)
Ad	0.27 (<0.05-0.77)
Yo	0.40 (0.05-1.50)
Ol	0.73 (<0.05-1.40)

CD20++CD21-CD24+ CD27-

Baseline cell counts in cells/µL	
Ch	0.55 (0.23-2.80)
Ad	0.18 (<0.05-0.96)
Yo	0.08 (<0.05-0.55)
Ol	0.24 (0.06-1.30)

CD20++CD21-CD24- CD27+

Baseline cell counts in cells/µL	
Ch	0.75 (0.14-4.80)
Ad	0.30 (<0.05-0.57)
Yo	0.28 (<0.05-0.75)
Ol	0.24 (<0.05-1.20)

CD20++CD21-CD24- CD27-

Baseline cell counts in cells/µL	
Ch	2.30 (0.75-7.10)
Ad	0.84 (0.12-4.30)
Yo	0.39 (0.17-3.20)
Ol	0.82 (0.16-2.10)

Figure S3. No significant changes in IgG1+ memory B-cell subsets upon vaccination. Over-time quantitative changes in IgG1+ memory B-cell subsets, presented as ratio over baseline (median, min-max). Dashed lines indicate a ratio over baseline of 0.67 and 1.5. Underneath each graph, the baseline cell counts per cohort are presented in cells/µL (median, min-max). Wilcoxon matched pair signed-ranked test followed by Bonferroni correction was used to assess differences in ratio compared to baseline over time. For longitudinal changes, only significant differences compared to baseline are shown. *, $p < 0.05$; **, $p \leq 0.01$; ***, $p \leq 0.001$.

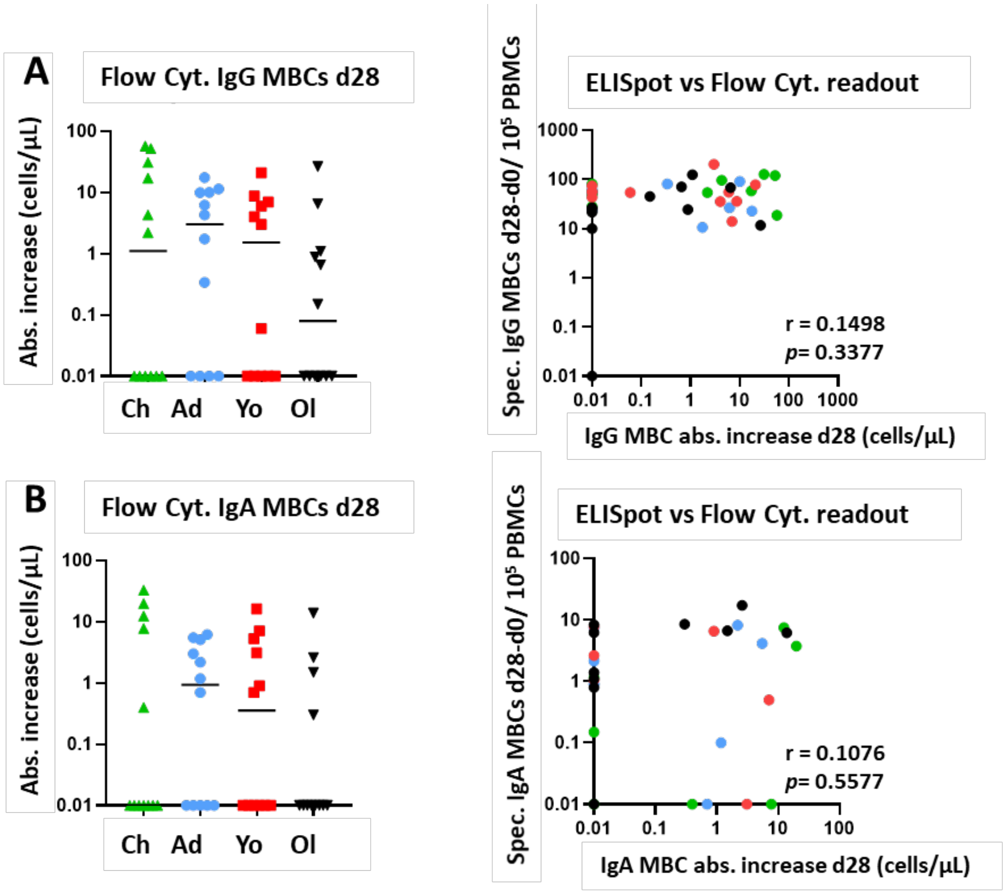

Figure S4. Correlation between cellular changes as measured by flow cytometry and ELISpot. (A) Left panel: expansion of IgG+ Memory B cells (day 28) per individual, expressed as absolute increase in cells/µL. Right panel: correlation between the ELISpot readings and the flow cytometry readout for IgG+ Memory B cells. **(B)** Left panel: expansion of IgA+ Memory B cells (day 28) per individual, expressed as absolute increase in cells/µL. Right panel: correlation between the ELISpot readings and the flow cytometry readout for IgA+ Memory B cells. Of note; for visualization purposes, all absolute increases lower than 0.01 were set to 0.01. The original values were used to calculate the Spearman Correlations. Flow Cyt. = flow cytometry; spec.= specific (in this case, specific for the tested vaccine antigens); MBC = Memory B cells; PBMC= peripheral blood mononuclear cells; d= days after vaccination; r= Spearman's Correlation coefficient; abs.= absolute.

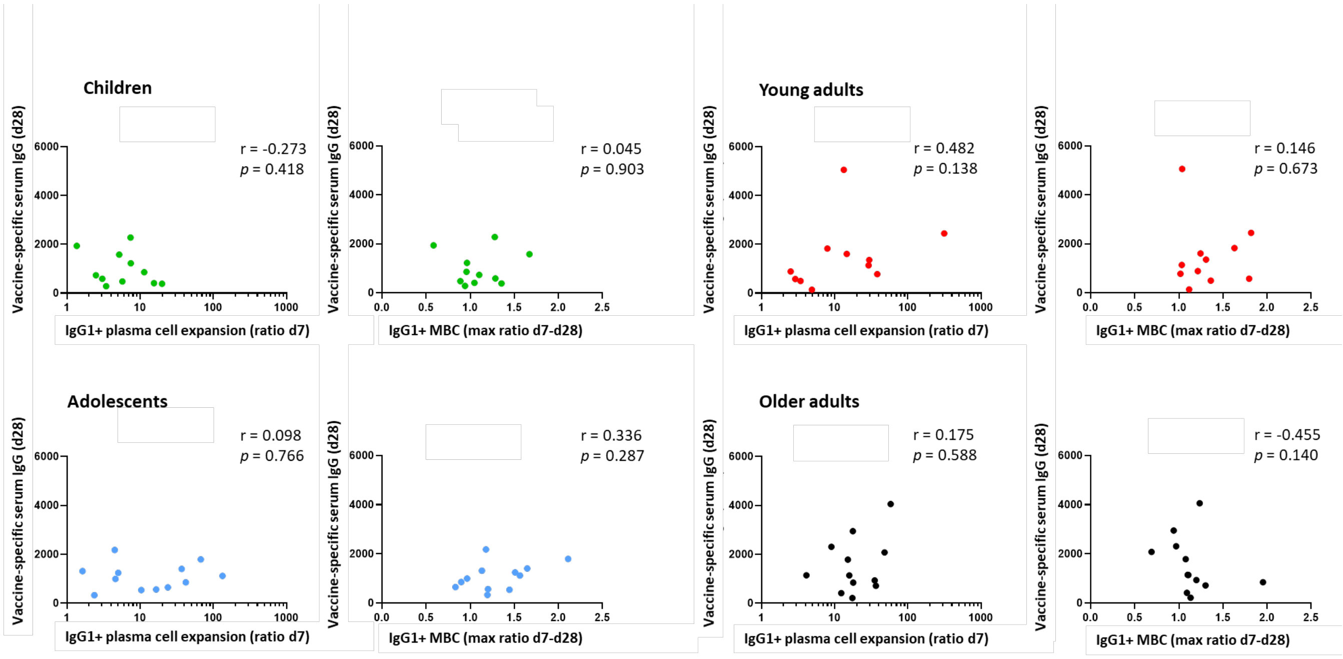

Figure S5. Correlation between cellular changes and the vaccine-specific serum IgG level post-vaccination as determined by Spearman's Ranking Correlation per age cohort. Per cohort the left plot shows the correlation between the maximum expansion of IgG1 plasma cells (day 7) and vaccine-specific serum IgG (directed against FHA, Prn, PT and Tet) (day 28). The right plots show the correlation between the maximum expansion of IgG1 memory B cells (day 14 or day 28) and vaccine-specific serum IgG (directed against FHA, Prn, PT and Tet) (day 28). MBC = memory B cell; r= Spearman's correlation coefficient; d= days after vaccination.

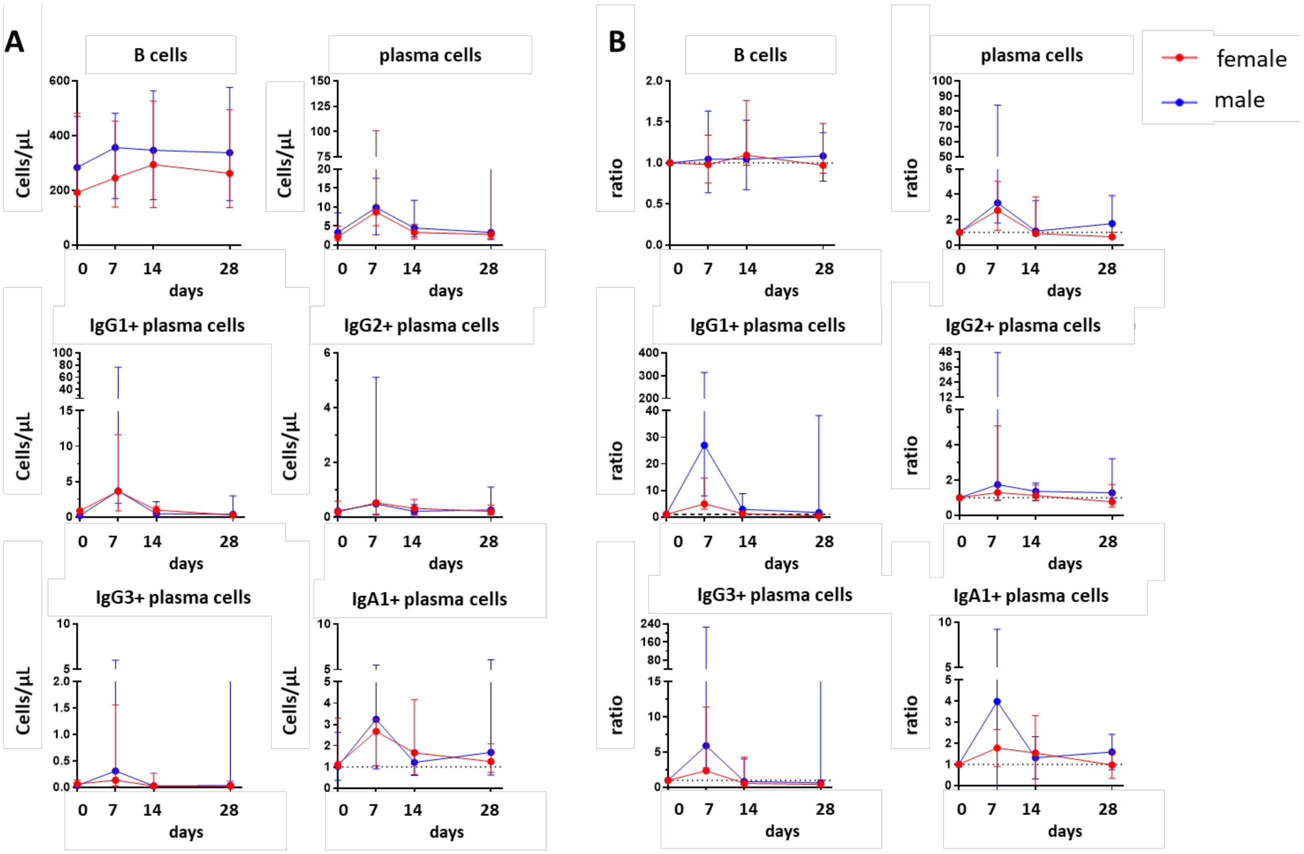

Figure S6. **Impact of sex on cellular responses after vaccination in the young adult cohort (all wP-primed).** Flow cytometry-derived cell numbers (absolute count in cells/µL (**A**) and ratio over baseline (**B**)) and their changes over time in an age-matched, wP-primed male (n= 7) and female (n= 5) cohort. Of note, for one male participant, no baseline B-cell data was available. Therefore, in the graphs showing the ratio over baseline, data of 6 males are shown, whereas absolute counts include the data of 7 males. Graphs indicate median + range. Dashed line indicates ratio of 1.0 (baseline value).

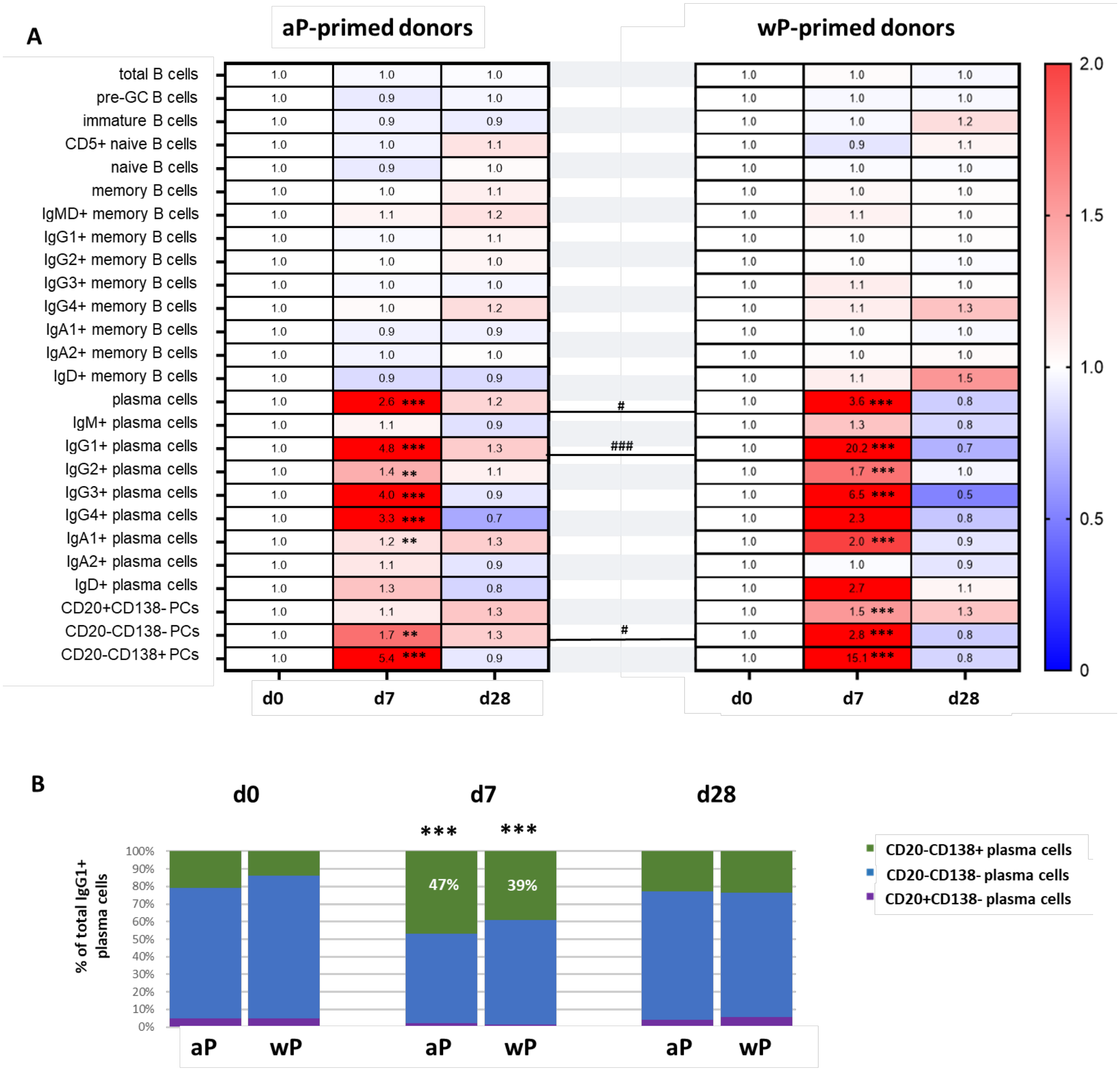

Figure S7. IgG1+ and total plasma cell expansion is more prominent in non-age-matched donors after wP priming. (A) Heatmap showing over-time changes in memory B-cell and plasma cell subsets in aP-primed (12 children + 5 adolescents) and wP-primed (7 adolescents and 12 young adults; not the older adults, because of their uncertain vaccination status) donors. **(B)** Over-time distribution of IgG1+ plasma cells representing different maturation stages with total IgG1+ plasma cells. Median values for each population were used to

construct the plots. Wilcoxon matched pair signed-ranked test followed by Bonferroni correction was used to assess longitudinal differences in percentage of CD20-CD138+ cells in total IgG1+ plasma cells within each cohort. Differences in the percentage CD20-CD138+ cells in total IgG1+ plasma cells between cohorts were assessed using Kruskal-Wallis followed by Dunn's test, but did not yield significant differences. For longitudinal changes, only significant differences compared to baseline are shown. Significant longitudinal differences within a cohort as indicated with ***, $p \leq 0.001$. Significant differences between cohorts at the same time point are indicated with #, $p < 0.05$; ###, $p \leq 0.001$. D= days after vaccination; aP = acellular pertussis vaccine; wP = whole cell pertussis vaccine.
