# Supplementary material for: Age and Primary Vaccination Background Influence the Plasma Cell Response to Pertussis Booster Vaccination
Source: Vaccines (Basel). 2022 Jan 18;10(2):136. doi: 10.3390/vaccines10020136 (PMC8878388; doi:10.3390/vaccines10020136)
Supplement: Supplementary file 1 [file vaccines-10-00136-s001.zip › Supplemental Table S1. Diks et al. B-cell kinetics after aP vaccination_proof prints.pdf]

Table S1. Complete overview of the inclusion and exclusion criteria for this study. This table is adjusted from a previous manuscript describing the inclusion and exclusion criteria of the total BERT study cohort (Versteegen, P., et al., EBioMedicine, 2021)[1].

Inclusion criteria

In order to be eligible to participate in this study, participants must meet all of the following criteria:

• normal general health;
• within the right age group for the cohort;
• received all regular vaccines for their age group according to the Dutch NIP in the Netherlands; a copy of the vaccination booklet will be included in the participant's documents. If booklet is not available for cohorts A, B and C, vaccination status will be checked with regulatory agencies / GP. For cohort C and D this booklet might not be available due to their age;
• provision of written informed consent from the adult participants and parents or legal guardians of minors;
• willing to adhere to the protocol and be available during the study period.

Exclusion criteria

Participants meeting any of the following criteria are excluded from participation in this study:

• present evidence of serious disease(s) within the last 3 months before inclusion requiring immunosuppressive or immune modulating medical treatment, such as systemic corticosteroids, that might interfere with the results of the study;
• chronic infection;
• known or suspected immune deficiency;
• history of any neurologic disorder, including epilepsy;
• previous administration of serum products (including immunoglobulins) within 6 months before vaccination and blood sampling;
• known or suspected allergy to any of the vaccine components (by medical history);
• occurrence of serious adverse events (SAEs) after primary DTwP-IPV vaccination, DTaP-IPV vaccination or any other vaccination (by medical history);
• vaccination with any pertussis containing vaccine other than those described in the inclusion criteria (i.e. only according to NIP);
• adult pertussis vaccination according to the NIP in the last 5 years (i.e. maternal vaccination);
• vaccination with any other diphtheria, tetanus or polio containing vaccine in the last 5 years, other than described in the NIP;
• children between 8 and 10 years of age eligible for cohort A who have already received the dT-IPV booster vaccination according to the Dutch NIP around 9 years of age;
• mixed wP and aP priming within a participant;
• pregnancy.

1. Versteegen, P., et al., *Responses to an acellular pertussis booster vaccination in children, adolescents, and young and older adults: A collaborative study in Finland, the Netherlands, and the United Kingdom.* 2021. **65**: p. 103247.
