# Supplementary material for: Age and Primary Vaccination Background Influence the Plasma Cell Response to Pertussis Booster Vaccination
Source: Vaccines (Basel). 2022 Jan 18;10(2):136. doi: 10.3390/vaccines10020136 (PMC8878388; doi:10.3390/vaccines10020136)
Supplement: Supplementary file 1 [file vaccines-10-00136-s001.zip › Supplemental Table S3. Diks et al. B-cell kinetics after aP vaccination_proof prints.pdf]

Table S3. Phenotypic descriptions used to define B-cell subsets stained with the EuroFlow B-cell panel by manual analysis. Of note: in this study, the automated gating and identification (AGI) module in the Infinicyt Software was used. The results obtained by the AGI-module are highly similar to, but more reproducible than, the results obtained by manual analysis. This AGI module makes use of clustering algorithms and comparison with fully annotated reference flow cytometry (FCS) data files of healthy individuals to assign clusters of events to a population. The removal of debris and doublets is not indicated in the analysis strategy below but should be performed to ensure high quality data.

Stepwise approach (gating in 2D plots)	Phenotypic description
#1. Identification of total plasma cells	CD45+CD19dimCD38highCD21-CD24-<br>Light scatter properties are low/medium (between lymphocytes and monocytes).
#2. Definition of maturation stage	• Least mature plasma cells: CD20+CD138-<br>• Intermediate mature plasma cells: CD20-CD138-<br>• Most mature plasma cells: CD20-CD138+
#3. Classification of plasma cells based on isotype	• IgM+, no expression of other isotype Igs<br>• IgG1+, no expression of other isotype Igs<br>• IgG2+, no expression of other isotype Igs<br>• IgG3+, no expression of other isotype Igs<br>• IgG4+, no expression of other isotype Igs<br>• IgA1+, no expression of other isotype Igs<br>• IgA2+, no expression of other isotype Igs<br>• IgD+, no expression of other isotype Igs<br>• IgH-, no Ig expression of any isotype Igs
#4. Classification of plasma cells based on CD62L expression	• CD62L-<br>• CD62L+
#5. Identification of total B cells	• CD45+CD19+CD20+ B cells show low light scatter characteristics (lymphocyte range)
#6. Identification of switched memory B-cell (MBC) subsets based on isotype. Switched MBCs express only one isotype	• IgG1+, no expression of other isotype Igs<br>• IgG2+, no expression of other isotype Igs<br>• IgG3+, no expression of other isotype Igs<br>• IgG4+, no expression of other isotype Igs<br>• IgA1+, no expression of other isotype Igs<br>• IgA2+, no expression of other isotype Igs
#7. Subclassification based on maturation/functional CD markers	• CD20+CD21+<br>o Homogenous CD24 staining<br>• CD20++CD21-/dim<br>o CD24+<br>o CD24-
#8. Subclassification based on CD62L/CD27 positivity	• CD27+CD62L+<br>• CD27+CD62L-<br>• CD27-CD62L-<br>• CD27-CD62L+

<table>
<tr><td>#9. Identification of non-switched MBCs</td><td>CD27+IgM++IgD+
Of note, a minor subset of IgD+IgM- MBCs may be found as well. These can be classified separately.</td></tr>
<tr><td>#10. Subclassification based on maturation/functional CD markers</td><td>CD20+CD21+
Homogenous CD24 staining
CD20++CD21-/dim
CD24+
CD24-
No further subclassification in these populations.</td></tr>
<tr><td>#11. Classification of pre-germinal center (preGC) B cells</td><td>CD27-IgM+IgD+</td></tr>
<tr><td>#12. Subclassification based on maturation/functional CD markers</td><td>Immature preGC B cells: CD38+CD24+CD5+CD21-/+
Naive CD5+ B cells: CD38-/dim CD24-/dimCD5+
Naive CD5- B cells:CD38-/CD24-/dimCD5-</td></tr>
<tr><td>#13. Subclassification of naive B cells based on maturation/functional CD markers</td><td>CD20+CD21+
Homogenous CD24 staining
CD20++CD21-/dim
CD24+
CD24-</td></tr>
</table>
