# Supplementary material for: Age and Primary Vaccination Background Influence the Plasma Cell Response to Pertussis Booster Vaccination
Source: Vaccines (Basel). 2022 Jan 18;10(2):136. doi: 10.3390/vaccines10020136 (PMC8878388; doi:10.3390/vaccines10020136)
Supplement: Supplementary file 1 [file vaccines-10-00136-s001.zip › Supplemental Table S4. Diks et al. B-cell kinetics after aP vaccination_proof prints.pdf]

Table S4. Baseline distribution of leukocytes, lymphocytes, T cells and NK cells (cells/µL, median (min-max)) in donor groups.

Cohort	Children			Adolescents			Young adults			Older adults		
age	7-10 y/o			11-15 y/o			20-34 y/o			60-70 y/o		
priming background	aP			mixed aP and wP			wP			no data available		
N=	12			12			12			12		
Cohort data	median	min.	max.	median	min.	max.	median	min.	max.	median	min.	max.
Leukocytes	5795	3397	14263	6148	3296	7837	5054	3224	6501	5234	3373	7547
Lymphocytes	2551	2128	3902	2389	1077	3312	2017	1699	3057	2003	1150	3080
T cells	1806	1132	3308	1674	814	2466	1481	1204	2405	1481	794	2312
NK cells	210	123	508	268	65	456	214	115	713	282	87	547

Reference values†	Children			Adolescents			Young adults / Older adults		
age	5-10 y/o			10-16 y/o			Adult cohort, no age range specified		
N=	35			23			51		
Reference data	median	5th Perc.	95th perc.	median	5th Perc.	95th perc.	median	5th Perc.	95th perc.
Lymphocytes	2800	1100	5900	2200	1000	5300	1800	1000	2800
T cells	1900	700	4200	1500	800	3500	1200	700	2100
NK cells	300	90	900	300	70	1200	30	90	600

†Source publication for the reference values: Comans-Bitter et al, Journal of Pediatrics, 1997, doi: 10.1016/s0022-3476(97)70200-2. When using the here-presented reference values, please refer to the source publication of this data.
